# Iron supplementation alleviates pathologies in a mouse model of facioscapulohumeral muscular dystrophy

**DOI:** 10.1172/JCI181881

**Published:** 2025-07-01

**Authors:** Kodai Nakamura, Huascar Pedro Ortuste Quiroga, Naoki Horii, Shin Fujimaki, Toshiro Moroishi, Keiichi I. Nakayama, Shinjiro Hino, Yoshihiko Saito, Ichizo Nishino, Yusuke Ono

**Affiliations:** 1 Department of Muscle Development and Regeneration, Institute of Molecular Embryology and Genetics,; 2 Department of Molecular and Medical Pharmacology, Faculty of Life Sciences, and; 3 Center for Metabolic Regulation of Healthy Aging, Faculty of Life Sciences, Kumamoto University, Kumamoto, Japan.; 4 Division of Cellular Dynamics, Medical Research Laboratory, and; 5 Anticancer Strategies Laboratory, Advanced Research Initiative, Institute of Integrated Research, Institute of Science Tokyo, Tokyo, Japan.; 6 Department of Molecular and Cellular Biology, Medical Institute of Bioregulation, Kyushu University, Fukuoka, Japan.; 7 Department of Medical Cell Biology, Institute of Molecular Embryology and Genetics, Kumamoto University, Kumamoto, Japan.; 8 Department of Neuromuscular Research, National Institute of Neuroscience, National Center of Neurology and Psychiatry, Tokyo, Japan.; 9 Muscle Biology Laboratory, Tokyo Metropolitan Institute for Geriatrics and Gerontology, Tokyo, Japan.

**Keywords:** Metabolism, Muscle biology, Muscle, Neuromuscular disease, Skeletal muscle

## Abstract

Facioscapulohumeral muscular dystrophy (FSHD) is a genetic muscle disease caused by ectopic expression of the toxic protein DUX4, resulting in muscle weakness. However, the mechanism by which DUX4 exerts its toxicity remains unclear. In this study, we observed abnormal iron accumulation in muscles of patients with FSHD and in mice with muscle-specific DUX4 expression (DUX4-Tg mice). Treatment with iron chelators, an iron-deficient diet, and genetic modifications inhibiting intracellular uptake of iron did not improve but rather exacerbated FSHD pathology in DUX4-Tg mice. Unexpectedly, however, iron supplementation, from either a high-iron diet or intravenous iron administration, resulted in remarkable improvement in grip strength and running performance in DUX4-Tg mice. Iron supplementation suppressed abnormal iron accumulation and the ferroptosis-related pathway involving increased lipid peroxidation in DUX4-Tg muscle. Muscle-specific DUX4 expression led to retinal vasculopathy, a part of FSHD pathology, which was prevented by iron administration. Furthermore, high-throughput compound screening of the ferroptosis pathway identified drug candidates including ferrostatin-1 (Fer-1), a potent inhibitor of lipid peroxidation. Treatment with Fer-1 dramatically improved physical function in DUX4-Tg mice. Our findings demonstrate that DUX4-provoked toxicity is involved in the activation of the ferroptosis-related pathway and that supplementary iron could be a promising and readily available therapeutic option for FSHD.

## Abstract 2

Our findings indicate that iron supplementation is a promising and readily available therapeutic option for the treatment of FSHD.

## Graphical abstract

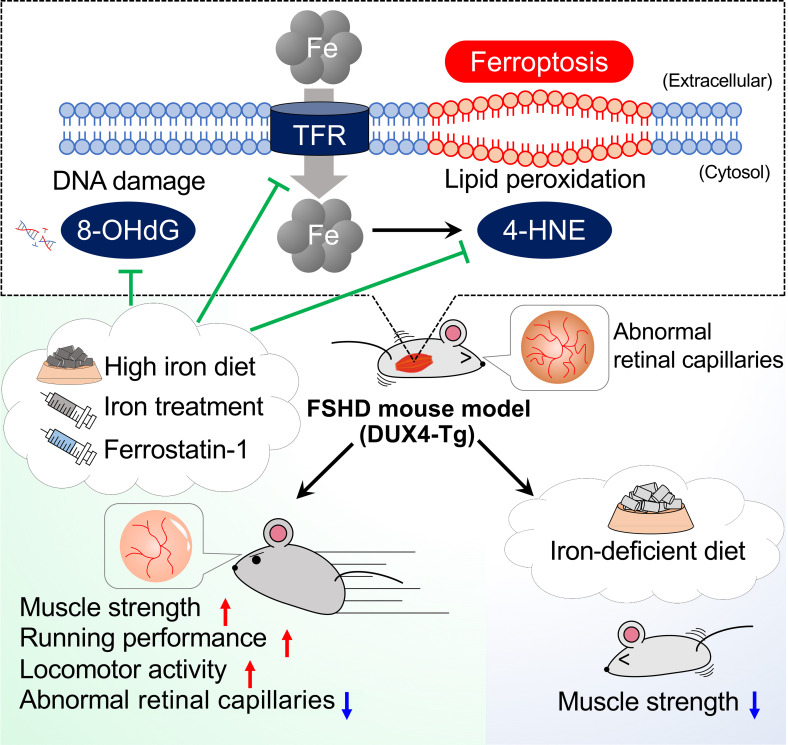

## Introduction

Facioscapulohumeral muscular dystrophy (FSHD), an autosomal dominant muscle disease, has no effective cure (1, 2). The disease is characterized by muscle weakness, starting with facial muscles, followed sequentially by the scapular stabilizer, upper arm, and lower leg muscles (3). In FSHD, muscle weakness is caused by aberrant expression of the full-length form of the transcription factor double homeobox 4 (DUX4) (1, 2, 4), whose expression is regulated by a complex genetic and epigenetic etiology. During the early phase of embryogenesis, DUX4 regulates germline genes involved in implantation (1–6). In adult tissues, the expression of DUX4 is epigenetically silenced in somatic cells, except in the testis and thymus. FSHD is associated with epigenetic derepression of the DUX4 gene, encoded by the D4Z4 macrosatellite repeat on the subtelomere region of chromosome 4q35 (1, 2, 7). DUX4 is a toxic protein that induces dystrophic alterations in the muscles. The current body of research has reached a consensus that DUX4 is the primary therapeutic target for FSHD (8–13). Although the mechanisms by which DUX4 exerts myotoxicity remain unclear, accumulating evidence has indicated that DUX4 induces oxidative stress, which plays an important role in FSHD pathogenesis (1, 2, 4, 14).

Fe^2+^ produces hydroxyl radicals, a highly active form of reactive oxygen species (ROS), via the Fenton reaction, leading to oxidative stress. Thus, iron metabolism is tightly regulated, and excess iron causes tissue and organ damage (15, 16). Abnormal iron metabolism in the muscles is associated with muscle diseases. Muscle iron levels increase with the dysregulation of iron-related proteins in *mdx* mice, a mouse model of Duchenne muscular dystrophy (DMD) (17, 18). Treatment with iron chelators reduces iron levels and oxidative stress and suppresses pathogenesis in *mdx* mice (17–19). Aberrant iron accumulation in muscles is also involved in the pathophysiology of age-related sarcopenia muscle atrophy (20, 21). Unlike in sarcopenia and DMD, iron insufficiency is observed in the majority of patients with cancer and is associated with a poor prognosis. Cancer cachexia is characterized by progressive muscle wasting in the late stages of cancer (22). A recent study has shown that iron supplementation improves cancer cachexia in tumor-bearing mice and muscle strength in patients with cancer (23). These results suggest that controlling iron homeostasis in muscles is important for maintaining muscle mass and regenerative ability. Whether the aberrant regulation of iron metabolism is implicated in FSHD has not yet been assessed.

In this study, we examined whether iron metabolism is related to FSHD pathogenesis in a mouse model of FSHD. We used mice with muscle-specific and tamoxifen-inducible (TMX)-inducible DUX4 expression (DUX4-Tg mice), which have recently been established as an FSHD mouse model (24, 25). We observed aberrant iron accumulation in the muscles of patients with FSHD and in DUX4-Tg mice. Unexpectedly, iron supplementation remarkably alleviated the pathophysiology in DUX4-Tg mice. Therefore, our findings provide a mechanism for DUX4-provoked toxicity and highlight a promising therapeutic approach for the treatment of FSHD.

## Results

### Patients with FSHD exhibited abnormal iron accumulation in muscles.

**Figure 1:**
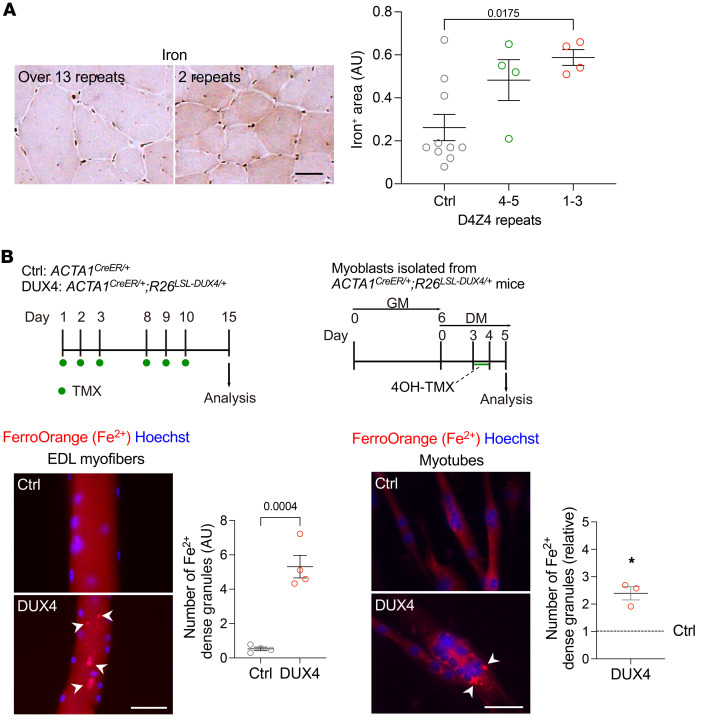
Abnormal iron accumulation in muscles of patients with FSHD and DUX4-Tg mice. (**A**) Iron staining in the muscle of FSHD patients using potassium ferricyanide (*n* = 4–10). (**B**) FerroOrange staining in EDL myofibers and culture myotubes. The EDL myofibers were isolated from control (Ctrl) and DUX4 mice given TMX (5 mg/kg body weight) three times per week for 2 weeks and immediately costained with FerroOrange and Hoechst. The number of FerroOrange-dense granules per myofiber was quantified (*n* = 4). Myoblasts isolated from *ACTA1^CreER/+^ R26^LSL-DUX4/+^* mice were induced to differentiate into myotubes in DM for 3 days, followed by 4-hydroxytamoxifen (4OH-TMX) treatment for 24 hours. Myotubes were then costained with FerroOrange and Hoechst on day 5 in DM. The number of FerroOrange-dense granules per myotube was quantified (*n* = 3). Arrowheads indicate iron-dense granules. Scale bars: 50 μm. Data represent the mean ± SEM. *P < 0.05; Student’s 2-tailed unpaired *t* test. *P* values were determined using 1-way ANOVA followed by Tukey’s multiple-comparison post hoc test.

In FSHD type 1 (FSHD1), which occurs in approximately 95% of patients with FSHD, contraction of the D4Z4 repeat number leads to chromatin relaxation and ectopic expression of DUX4 in the muscles (1, 2). The number of D4Z4 unit repeats is correlated with disease severity in FSHD1, with carriers of 1–6 repeats being more severely affected (26, 27). Patients with 1–3 repeats show earlier onset and greater disease severity in muscle and non-muscle symptoms, such as hearing loss and retinal vascular vasculopathy, whereas patients with 4–7 repeats showed more moderate disease manifestations (26, 27). To investigate the intramuscular iron levels in patients with FSHD, muscle cross sections were stained for iron. We analyzed 8 samples with 1–5 D4Z4 repeats from patients with FSHD. To represent a control group, we used samples from individuals who had over 13 D4Z4 repeats with some medical symptoms but did not show any obvious muscle pathologies. Histochemical analysis revealed that iron accumulated at a higher level in patients with FSHD than in controls (Figure 1A and Supplemental Figure 1; supplemental material available online with this article; https://doi.org/10.1172/JCI181881DS1).

We examined whether DUX4 expression altered intracellular iron levels in the mouse muscle. We used TMX-inducible DUX4-Tg mice by crossing *ACTA1^CreER/+^* mice (also known as HSA-MCM; ref. 24) with *R26^LSL-DUX4^* mice (25). To induce the expression of DUX4 in myofibers, TMX was intraperitoneally injected 3 times per week for 2 weeks into *ACTA1^CreER/+^ R26^LSL-DUX4/+^* mice. Individual myofibers were isolated from the extensor digitorum longus (EDL) muscle as previously described (28) and stained with FerroOrange, a highly sensitive fluorescent probe to detect Fe^2+^ in living cells, immediately after isolation (Figure 1B). Although we observed blurry spread autofluorescence background staining of FerroOrange, the FerroOrange^+^ dense granules were clearly detected in DUX4-Tg myofibers. We thus measured the granularities instead of showing the average fluorescence intensity of FerroOrange staining throughout the myofibers and confirmed a greater amount of granulated Fe^2+^ in DUX4-Tg myofibers and myotubes (Figure 1B). These results indicate that DUX4 expression causes abnormalities in iron metabolism in muscle.

### Iron insufficiency attenuated intracellular iron levels but mitigated muscle dysfunction in DUX4-Tg mice.

**Figure 2:**
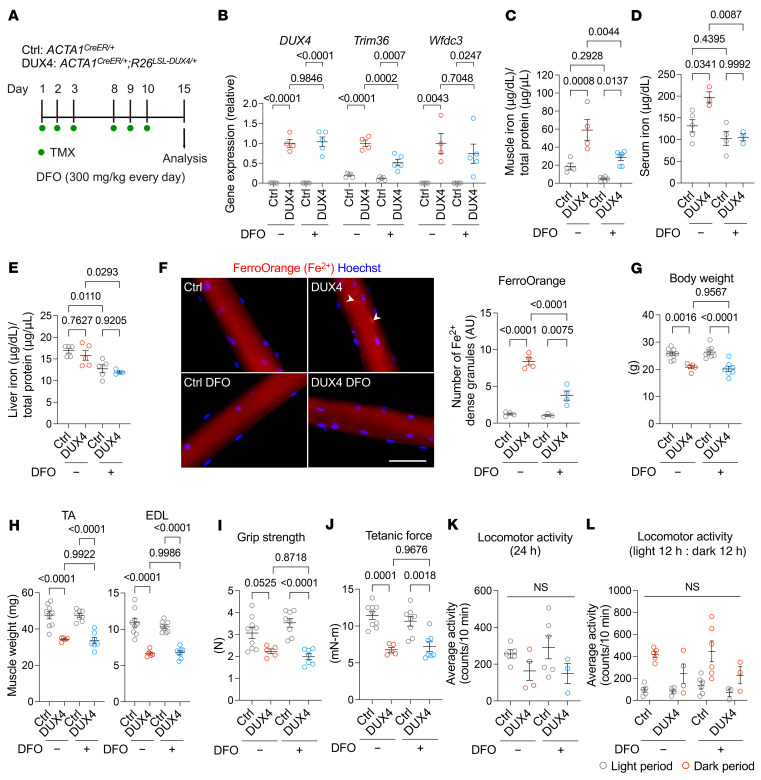
Iron chelator effects on DUX4-Tg mice. (**A**) TMX (5 mg/kg body weight) was intraperitoneally injected into control and DUX4-Tg mice 3 times a week for 2 weeks. The EDL myofibers were isolated from mice and immediately costained with FerroOrange and Hoechst. Deferoxamine (DFO; 300 mg/kg body weight) was intraperitoneally injected every day. PBS was used as control. (**B**) qPCR analysis of *DUX4* and target genes (*Trim36* and *Wfdc3*) in quadriceps muscle (*n* = 4–5). (**C**–**E**) Iron contents in muscle, serum, and liver (*n* = 3–6). (**F**) FerroOrange staining in EDL myofibers (*n* = 3–4). (**G**) Body weight (*n* = 5–9). (**H**) Muscle weights (*n* = 5–9). (**I**) Grip strength (*n* = 5–9). (**J**) Tetanic muscle force (*n* = 5–9). (**K** and **L**) Locomotor activity (*n* = 3–6). Arrowheads indicate iron-dense granules. Scale bar: 50 μm. Data represent the mean ± SEM. *P* values were determined using 2-way ANOVA followed by Tukey’s multiple-comparison post hoc test.

We next investigated the effect of iron insufficiency in DUX4-Tg mice in vivo. To test whether iron chelators suppress the cellular toxicity of DUX4 in muscle, the iron chelator deferoxamine (DFO; 300 mg/kg) was intraperitoneally administered into DUX4-Tg mice daily for 2 weeks (Figure 2A). Quantitative real-time PCR (qPCR) analysis revealed that *DUX4* and its target genes (*Trim36* and *Wfdc3*) were upregulated in DUX4-Tg muscles with or without DFO, whereas the expression of *Trim36* in DFO-treated mice was slightly lower than that in DFO-untreated mice (Figure 2B). The total iron contents were measured by iron colorimetric assay. We found that the iron levels were remarkably upregulated in muscle and serum, but not in liver, in DUX4-Tg mice, whose upregulations were suppressed by DFO (Figure 2, C–E). Consistent with these observations, treatment with DFO effectively reduced the amount of granulated iron in DUX4-Tg myofibers (Figure 2F). These data indicate that increased levels of local iron granularity with FerroOrange staining are a hallmark of excess iron accumulation in muscle. Despite reduced iron levels, we found that the expression of DUX4 in muscle resulted in a decrease in body weight (Figure 2G), muscle weight (Figure 2H), grip strength (Figure 2I), and muscle force generation (Figure 2J). Voluntary locomotor activity remained unchanged following DUX4 induction (Figure 2, K and L). Similarly, treatment with another iron chelator, deferasirox (DFX), which has a longer half-life than DFO and is more stable, did not improve muscle function or muscle weight, whereas granulated iron levels decreased in DFX-treated EDL myofibers (Supplemental Figure 2, A–I).

A key iron sensor, iron regulatory protein 2 (IRP2), controls iron homeostasis by binding to iron-responsive elements (IREs) in mRNAs encoding iron metabolism–related proteins, such as transferrin receptor (TFR) and ferritin (29). Under iron-deficient conditions, IRP2 binds to IREs to facilitate the intracellular iron uptake by post-transcriptionally controlling mRNA stability and translation. To examine the effect of IRP2 inactivation in DUX4-Tg mice, we generated *Irp2^–/–^* DUX4-Tg mice by crossing an *Irp2*-deficient mouse line (29, 30) with an *ACTA1^CreER/+^ R26^LSL-DUX4^* mouse line (Supplemental Figure 3A). While *DUX4* and its target genes *Trim36* and *Wfdc3* were upregulated in DUX4-Tg mice, the expression levels of *Wfdc3*, but not *DUX4* or *Trim36*, in *Irp2^–/+^* DUX4-Tg and *Irp2^–/–^* DUX4-Tg mice were slightly lower than those in DUX4-Tg mice (Supplemental Figure 3B). Although the level of iron accumulation was reduced upon IRP2 inactivation (Supplemental Figure 3C), *Irp2* deficiency did not improve body weight, grip strength, muscle force generation, or muscle weight in DUX4-Tg mice (Supplemental Figure 3, D–G). Treadmill running performance was remarkably impaired following the induction of DUX4 but was not improved upon *Irp2* inactivation (Supplemental Figure 3H).

**Figure 3:**
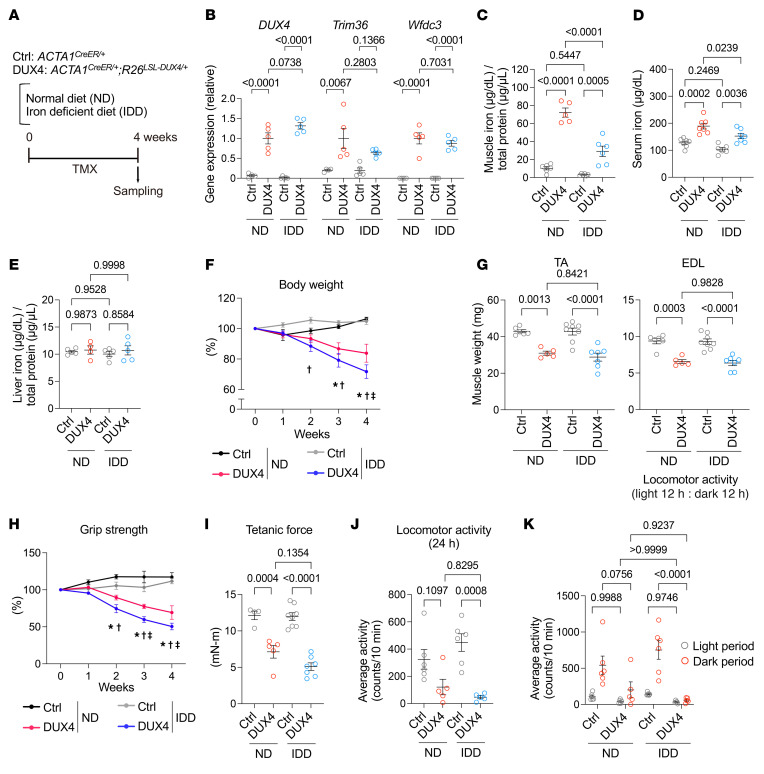
Effects of iron-deficient diet on DUX4-Tg mice. (**A**) Control and DUX4-Tg mice were fed ND or iron-deficient diet (IDD) mixed with TMX at 0.03 mg/g feed for 4 weeks. (**B**) qPCR analysis of *DUX4* and its target genes in quadriceps muscle (*n* = 4–5). (**C**–**E**) Iron contents in muscle, serum, and liver (*n* = 4–7). (**F**) Body weight (*n* = 5–8). (**G**) Muscle weights (*n* = 5–8). (**H**) Grip strength (*n* = 5–8). (**I**) Tetanic muscle force (*n* = 4–8). (**J** and **K**) Locomotor activity (*n* = 5–6). Data represent the mean ± SEM. *P* values were determined using 2-way ANOVA followed by Tukey’s multiple-comparison post hoc test. *P < 0.05, control ND vs. DUX4 ND; ^†^*P* < 0.05, control IDD vs. DUX4 IDD; ^‡^*P* < 0.05, DUX4 ND vs. DUX4 IDD.

We further examined the effect of iron insufficiency induced by an iron-deficient diet (IDD) on DUX4-Tg mice (Figure 3A). A standard normal diet (ND) was used as the control. Mice were fed either IDD or ND in powdered form containing TMX at a concentration of 0.03 mg/g feed for 4 weeks. The IDD did not affect the expression of DUX4 or its target genes in DUX4-Tg mice (Figure 3B). Consistent with observations in the DFO-treated condition, iron colorimetric assay revealed that the total iron contents were decreased in muscle and serum, but not in liver, by IDD in DUX4-Tg mice (Figure 3, C–E). Forced expression of DUX4 in the muscles resulted in a remarkable reduction in all parameters, including body weight (Figure 3F), muscle weight (Figure 3G), grip strength (Figure 3H), muscle force generation (Figure 3I), and voluntary locomotor activity (Figure 3, J and K) under both ND and IDD feeding conditions after TMX administration.

Altogether, our results indicate that iron insufficiency models (iron chelators, IRP2 deletion, and IDD) all attenuated intramuscular iron levels, albeit with no beneficial effect on DUX4-Tg mice, which suffered physical function deterioration.

### Iron supplementation alleviated physical function in DUX4-Tg mice.

**Figure 4:**
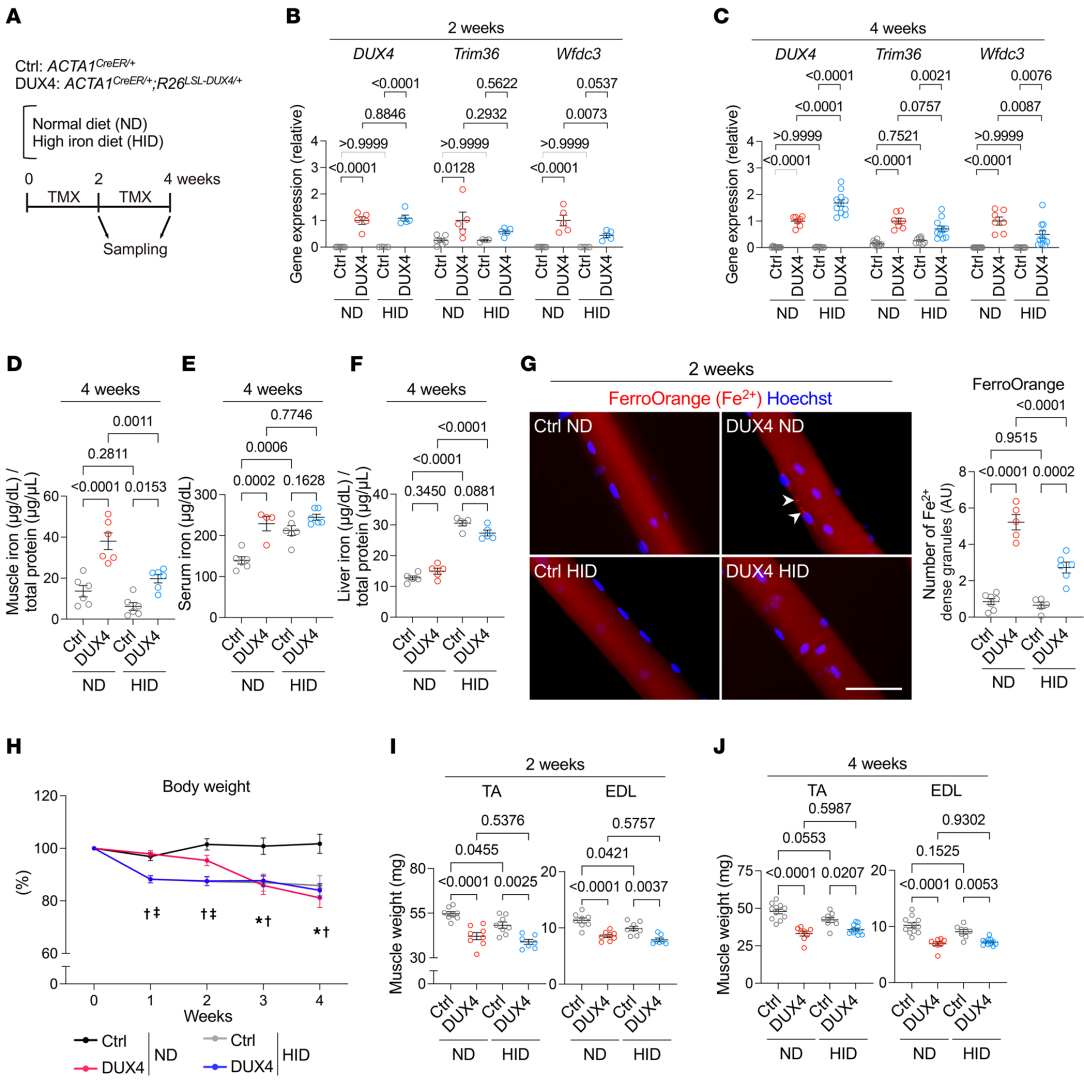
Effects of high-iron diet on DUX4-Tg mice. (**A**) Control and DUX4-Tg mice were fed ND or HID mixed with TMX at 0.03 mg/g feed for 2 or 4 weeks. (**B** and **C**) qPCR analysis of *DUX4* and its target genes in quadriceps muscle at 2 (*n* = 4–7) or 4 (*n* = 7–11) weeks. (**D**–**F**) Iron contents in muscle, serum, and liver (*n* = 4–6). (**G**) FerroOrange staining in EDL myofibers (*n* = 5–7). (**H**) Body weight (*n* = 8–18). (**I** and **J**) Muscle weights at 2 (*n* = 7–8) or 4 (*n* = 8–11) weeks. Arrowheads indicate iron-dense granules. Scale bar: 50 μm. Data represent the mean ± SEM. *P* values were determined using 2-way ANOVA followed by Tukey’s multiple-comparison post hoc test. *P < 0.05, control ND vs. DUX4 ND; ^†^*P* < 0.05, control ND vs. control HID; ^‡^*P* < 0.05, DUX4 ND vs. DUX4 HID.

Having shown that iron insufficiency exacerbated the DUX4-provoked physical dysfunction, we examined the effect of iron supplementation on DUX4-Tg mice by feeding a high-iron diet (HID). The HID and ND were fed in a powdered form containing TMX at a concentration of 0.03 mg/g feed for 2 or 4 weeks (Figure 4A). qPCR analysis revealed that *DUX4* was similarly upregulated by TMX in mice fed ND or HID at 2 weeks; however, *DUX4* levels were higher in HID-fed DUX4-Tg mice than in ND-fed DUX4-Tg mice at 4 weeks (Figure 4, B and C). The expression levels of the DUX4 target genes *Trim36* and *Wfdc3* were slightly lower in the HID group than in the ND group at 2 and 4 weeks (Figure 4, B and C). Iron colorimetric assay revealed that HID reduced the total iron contents in the muscle tissue of DUX4-Tg mice, while iron levels in serum and liver were increased by HID (Figure 4, D–F). These results suggest that regulation in iron metabolism differs among muscle, serum, and liver. FerroOrange staining confirmed that iron accumulation in isolated myofibers was reduced under HID conditions (Figure 4G). HID feeding reduced the body weight of both DUX4-Tg and control mice in the first week after feeding (Figure 4H). This body weight loss occurred faster than that in DUX4-Tg mice fed ND (Figure 4H), probably because the taste of iron caused loss of appetite, affecting the amount of feeding. Muscle weight also decreased in both ND- and HID-fed DUX4-Tg mice (Figure 4, I and J).

**Figure 5:**
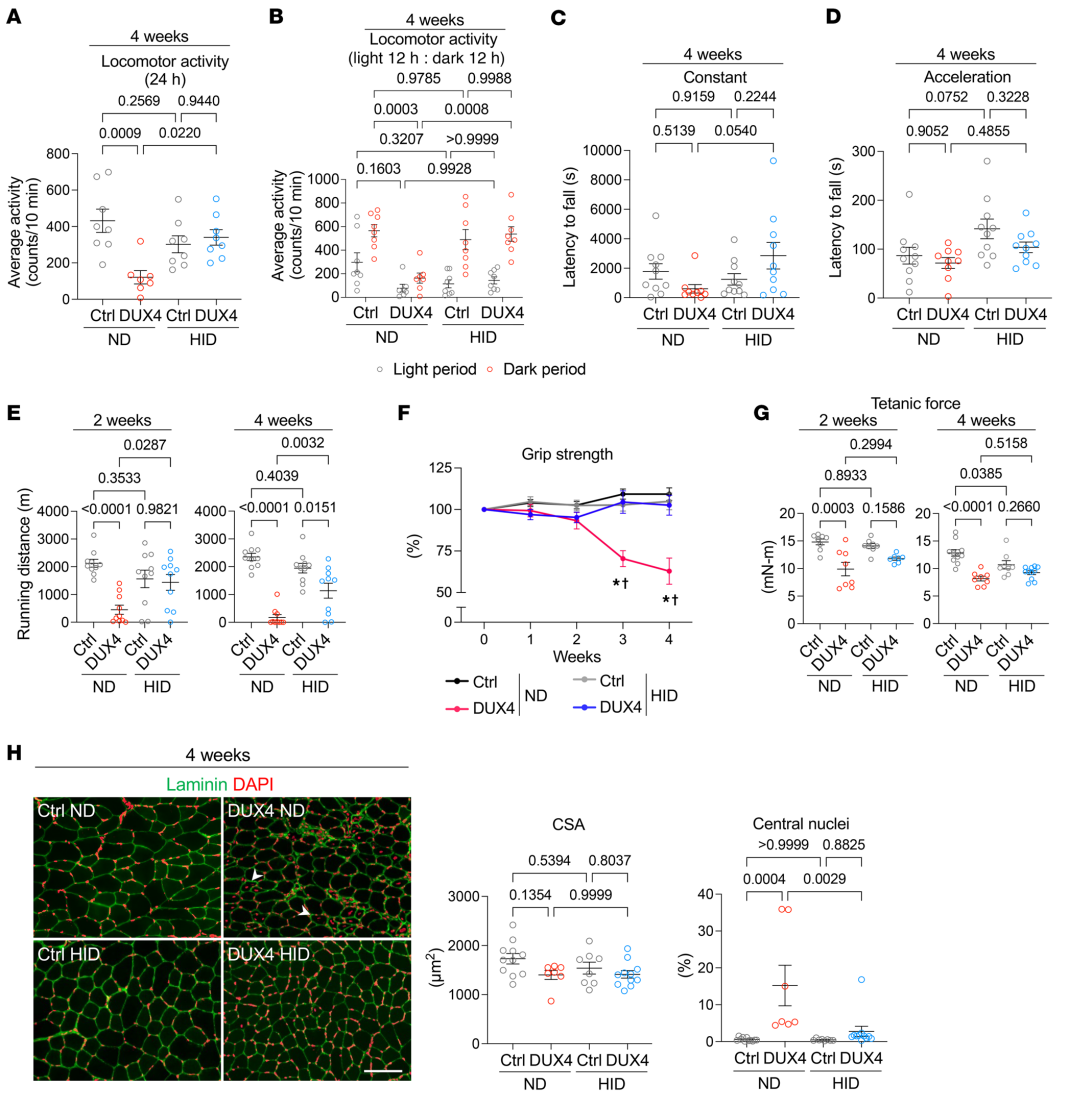
High-iron diet alleviates pathologies in DUX4-Tg mice. Control and DUX4-Tg mice were fed ND or HID mixed with TMX at 0.03 mg/g feed as shown in Figure 4. (**A** and **B**) Locomotor activity (*n* = 7–8). (**C** and **D**) Rotarod test (*n* = 9–10). (**E**) Running test (*n* = 10). (**F**) Grip strength (*n* = 8–18). (**G**) Tetanic muscle force at 2 (*n* = 7–8) or 4 (*n* = 8–11) weeks. (**H**) Immunohistochemistry for laminin to measure the cross-sectional area (CSA) and the percentage of myofibers with centrally located nuclei in tibialis anterior (TA) muscles (*n* = 7–11). Arrowheads indicate centrally nucleated myofibers. Scale bar: 100 μm. Data represent the mean ± SEM. *P* values were determined using 2-way ANOVA followed by Tukey’s multiple-comparison post hoc test. *P < 0.05, control ND vs. DUX4 ND; ^†^*P* < 0.05, DUX4 ND vs. DUX4 HID.

Subsequently, we examined the effect of iron supplementation on the physical functions of DUX4-Tg mice. Interestingly, iron supplementation ameliorated voluntary locomotor activity upon HID feeding compared with that upon ND feeding in DUX4-Tg mice (Figure 5, A and B). Although there was no change in the rotarod test results between the ND and HID groups in DUX4-Tg mice (Figure 5, C and D), treadmill running performance was remarkably improved following HID feeding at both 2 and 4 weeks (Figure 5E). More strikingly, HID completely prevented the DUX4-induced decline in grip strength in DUX4-Tg mice (Figure 5F). These data suggest that iron supplementation effectively prevents physical dysfunction in DUX4-Tg mice.

The HID itself seemed to suppress muscle force generation even in control mice at 4 weeks, but no difference in muscle force generation was observed between control and DUX4-Tg mice under HID conditions (Figure 5G). The cross-sectional area of the tibialis anterior (TA) muscle was unchanged among the groups, while HID markedly reduced the proportion of myofibers containing the central nucleus, which is a hallmark of regenerative myofibers (Figure 5H), suggesting that iron supplementation prevents DUX4-induced muscle damage. In support of this finding, the levels of serum creatine kinase, a marker of muscle damage (31), increased in ND-fed DUX4-Tg mice but not under HID conditions (Supplemental Figure 4). In addition to the standard ND, we evaluated another standard normal diet (ND2) as an alternative control, which was a synthetic diet with composition identical to the IDD and HID (except for iron content). We confirmed that ND and ND2 were comparable in gene expression profiles, body and muscle weights, and grip strength in DUX4-Tg mice (Supplemental Figure 5, A–E).

**Figure 6:**
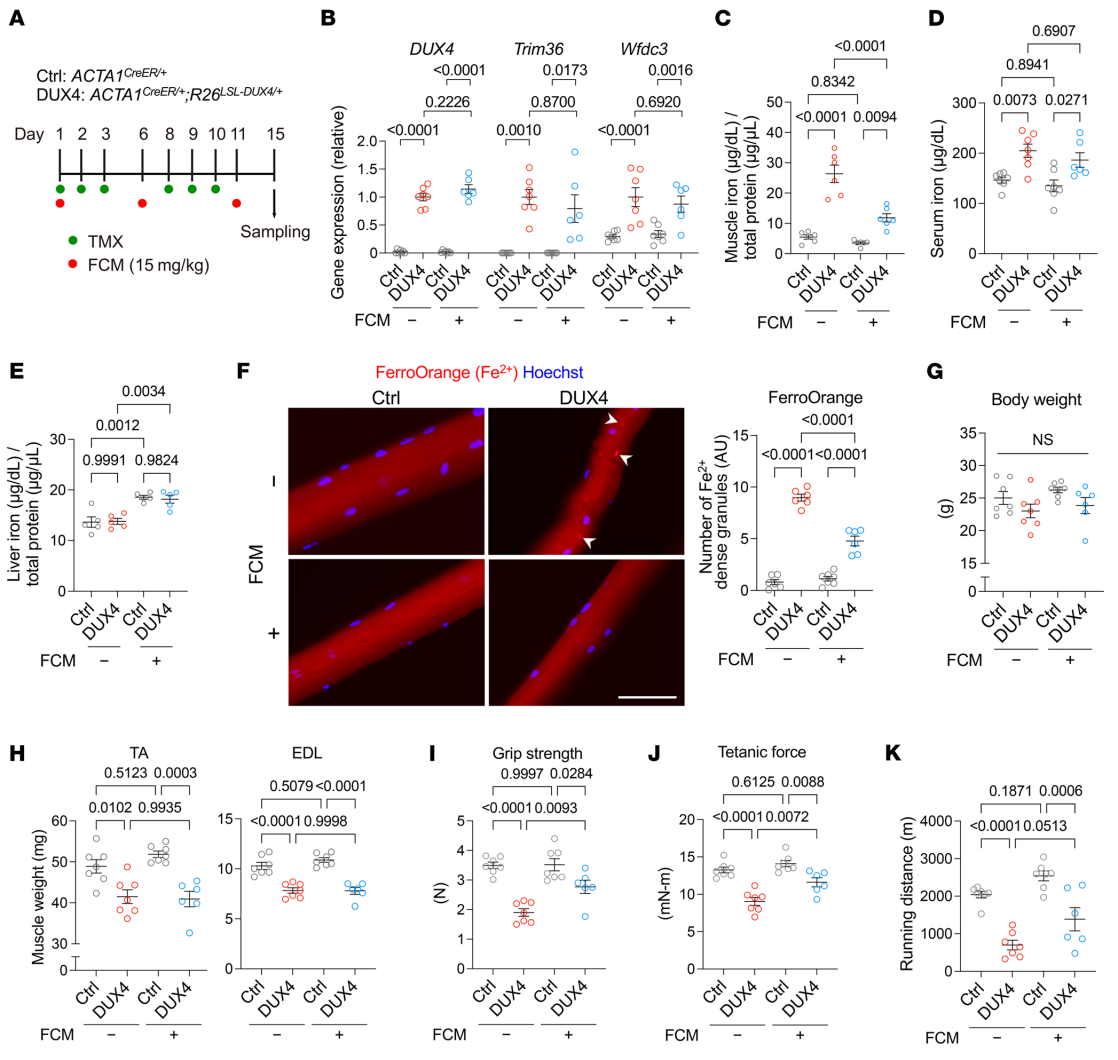
Intravenous iron administration ameliorates pathologies in DUX4-Tg mice. (**A**) TMX (5 mg/kg body weight) was intraperitoneally injected into control or DUX4-Tg mice 3 times per week for 2 weeks. Ferric carboxymaltose (FCM; 15 mg/kg body weight) was injected into the tail vein every 5 days. (**B**) qPCR analysis of *DUX4* and its target genes in quadriceps muscle (*n* = 6–7). (**C**–**E**) Iron contents in muscle, serum, and liver (*n* = 5–7). (**F**) FerroOrange staining in EDL myofibers (*n* = 6–7). Arrowheads indicate iron-dense granules. Scale bar: 50 μm. (**G**) Body weights (*n* = 6–7). (**H**) Muscle weights (*n* = 6–7). (**I**) Grip strength (*n* = 6–7). (**J**) Tetanic muscle force (*n* = 6–7). (**K**) Running test (*n* = 6–7). Data represent the mean ± SEM. *P* values were determined using 2-way ANOVA followed by Tukey’s multiple-comparison post hoc test.

The HID effectively suppressed the DUX4-provoked physical dysfunction in DUX4-Tg mice. However, it was not possible to determine an accurate amount of iron supplementation to prevent DUX4 toxicity using our feeding method. Considering this clinical implication, we tested the effect of iron administration using ferric carboxymaltose (FCM), an FDA-approved drug for patients with anemia. The FCM administration protocol was based on a previous study (23). As described in Figure 2, DUX4 was induced by the intraperitoneal injection of TMX in *ACTA1^CreER/+^ R26^LSL-DUX4^* mice, and 15 mg/kg FCM was administered every 5 days via tail vein injection (Figure 6A). qPCR analysis revealed that *DUX4* and its target genes were similarly upregulated in DUX4-Tg mice with or without FCM (Figure 6B). Consistent with the observations for the HID (Figure 4, D–F), the total iron contents were reduced in muscle, but not in serum and liver, in DUX4-Tg mice by FCM (Figure 6, C–E). FCM treatment also attenuated DUX4-induced iron accumulation in isolated myofibers (Figure 6F). Although body weight was unaltered among the groups (Figure 6G), forced expression of DUX4 resulted in a reduction in muscle weight in FCM-treated and untreated mice (Figure 6H). The administration of FCM ameliorated the decrease in grip strength of DUX4-Tg mice (Figure 6I) and muscle force generation (Figure 6J). Treadmill running performance tended to improve with the administration of FCM (Figure 6K). Thus, our results suggest that prolonged iron supplementation via oral and intravenous administration exerts beneficial effects on physical function in DUX4-Tg mice.

### Upregulation of inflammatory and lysosomal genes in DUX4-Tg muscles was repressed by iron supplementation.

**Figure 7:**
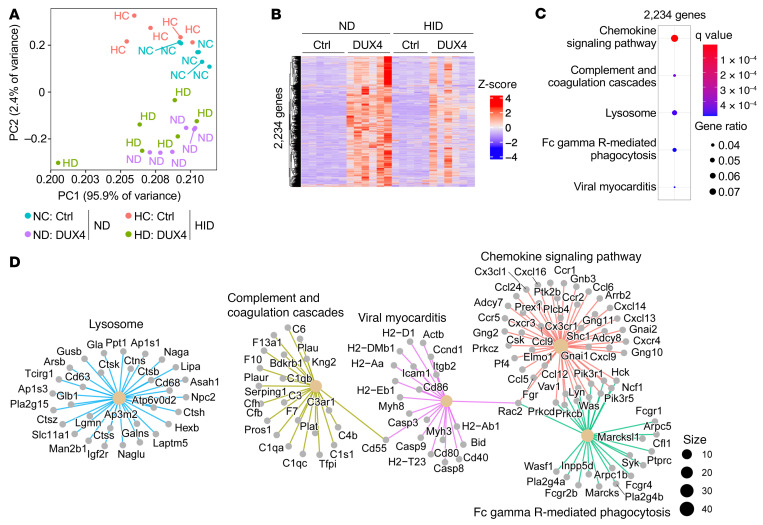
Upregulated genes in DUX4-Tg muscles and the effects of iron supplementation. (**A**–**D**) Transcriptome analysis of gastrocnemius and plantaris muscles in control and DUX4-Tg mice fed ND or HID mixed with TMX at a concentration of 0.03 mg/g feed for 4 weeks as shown in Figure 4A. Principal component analysis (PCA) plots, heatmaps, and enrichment analysis for each pattern of variation were created using the following RNAseqChef thresholds: fold change > 1.2, FDR < 0.05, and base mean = 0. (**A**) PCA plot (*n* = 5–6). (**B**) Heatmap of 2,234 genes that were highly upregulated specifically in DUX4-Tg mice fed ND compared with DUX4-Tg mice fed HID (*n* = 5–6). (**C** and **D**) Enrichment analysis (*n* = 5–6).

We performed transcriptome analysis using RNA sequencing (RNA-Seq) to visualize altered genes in the gastrocnemius and plantaris muscles of DUX4-Tg mice between the ND and HID conditions 4 weeks after TMX administration (Figure 7A). We identified 2,234 genes (fold change > 1.2, *q* value < 0.05) that were highly upregulated specifically in DUX4-Tg mice fed ND compared with those in DUX4-Tg mice fed HID and those in other control groups (Figure 7B). Immune system abnormalities have been reported in the muscles of patients with FSHD (12, 32). Enrichment analysis based on the Kyoto Encyclopedia of Genes and Genomes showed that these upregulated genes were associated with immune system–related pathways such as chemokine signaling and lysosomal proteolysis (Figure 7, C and D). Conversely, 2,018 genes (fold change > 1.2, *q* value < 0.05) were identified as downregulated specifically in the ND-feeding DUX4-Tg group compared with the HID-fed DUX4-Tg group as well as other control groups, which included insulin signaling and muscle contraction pathways (Supplemental Figure 6, A–C).

### DUX4 activated the ferroptosis-related pathway, which was suppressed by iron supplementation.

**Figure 8:**
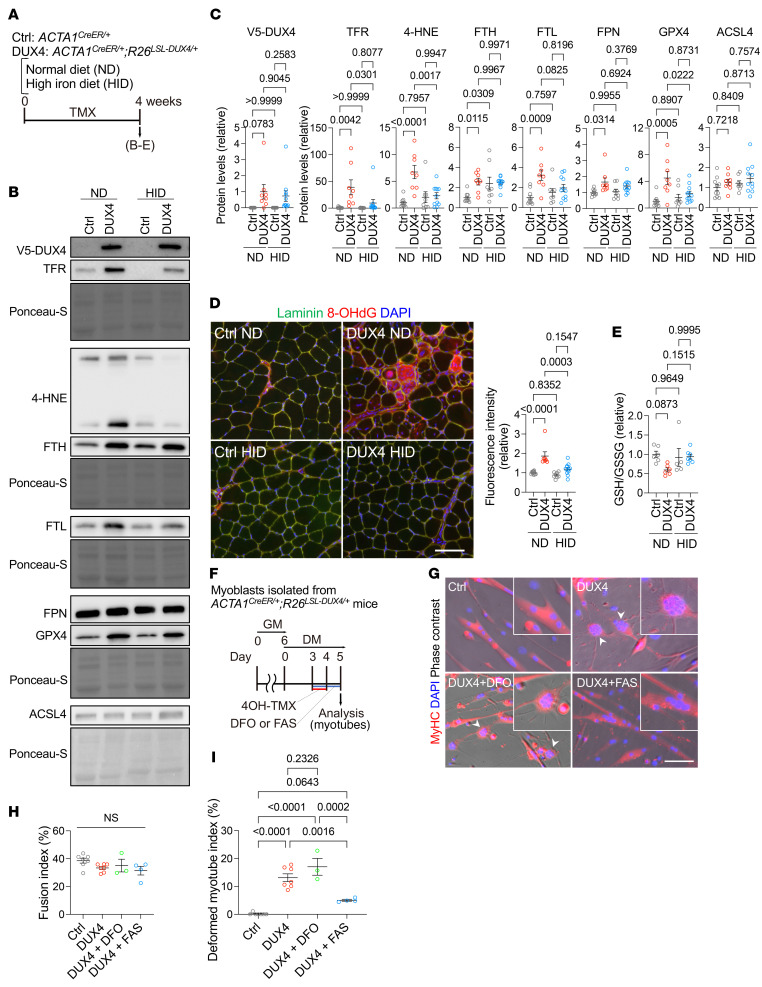
Iron supplementation suppresses DUX4-activated ferroptosis-related pathway. (**A**) Time course. Control or DUX4-Tg mice were fed ND or HID mixed with TMX at a concentration of 0.03 mg/g feed for 4 weeks as shown in Figure 4. (**B** and **C**) Immunoblot analysis for protein expression in quadriceps muscles (*n* = 8–11). (**D**) Immunohistochemistry for 8-OHdG and laminin to measure DNA damage in TA muscle (samples also used in Figure 5H). Scale bar: 100 μm; *n* = 7–11. (**E**) GSH/GSSG assay of biceps muscles (*n* = 5–7). (**F**–**I**) Myoblasts were induced to differentiate into myotubes in culture as shown in Figure 1B. Cultured myotubes were treated with 20 μM DFO or 10 μM FAS for 48 hours in DM. Morphological analysis determined fusion index (**H**) and deformed myotube index (**I**). Arrowheads indicate deformed myotubes. Scale bar: 100 μm; *n* = 3–7. Data represent the mean ± SEM. *P* values were determined using 2-way or 1-way ANOVA followed by Tukey’s multiple-comparison post hoc test.

Our findings indicated that iron supplementation exerts favorable effects on DUX4-expressing muscles, accompanied by reduced aberrant iron accumulation. These unexpected results prompted us to investigate how DUX4 toxicity is attenuated by iron supplementation. Ferroptosis is a programmed form of iron-induced cell death that involves the accumulation of lipid peroxidation, resulting in tissue and organ damage distinct from apoptosis, necrosis, and autophagy (33). Recent studies have implicated ferroptosis in a variety of diseases and pathologies in humans (34); however, no studies have reported on the involvement of the ferroptosis pathway in FSHD muscles. We thus aimed to determine whether the ferroptosis pathway is associated with DUX4-provoked cell toxicity under ND and HID conditions (Figure 8A). The DUX4 protein levels were consistent between both groups (Figure 8, B and C). We found that the levels of 4-hydroxynonenal (4-HNE), a marker of lipid peroxidation (35), and TFR protein were highly upregulated by DUX4, which was markedly suppressed upon HID feeding (Figure 8, B and C). Ferritin is composed of a polymer of ferritin heavy chain (FTH) and ferritin light chain (FTL), which regulate iron metabolism by storing and transporting iron (36). Ferroportin1 (FPN) is a nonheme cellular iron exporter. We showed that ACSL4, which regulates ferroptosis sensitivity by shaping the cellular lipid composition (37, 38), was not altered among the groups, but FTH, FTL, and FPN were upregulated upon DUX4 induction under the ND condition. Glutathione peroxidase 4 (GPX4) is a major antioxidant enzyme that prevents lipid hydroperoxidation and consequently ferroptosis (39). GPX4 was upregulated only in DUX4-Tg mice but suppressed by HID (Figure 8, B and C). Immunohistochemistry revealed a marked upregulation of the oxidative DNA damage biomarker 8-OHdG in DUX4-Tg muscles, which was suppressed upon iron supplementation (Figure 8D). Glutathione status (reduced glutathione [GSH]/oxidized glutathione [GSSG]) tended to be reduced in the muscle of DUX4-Tg mice, which was improved by iron supplementation (Figure 8E). These findings suggest that iron metabolism is dysregulated in muscles expressing DUX4, resulting in the accumulation of intramuscular iron, which may activate the ferroptosis-related pathway.

We demonstrated that iron supplementation remarkably ameliorated muscle pathology in DUX4-Tg mice in vivo. However, whether supplementary iron directly inhibits DUX4-provoked myotoxicity remains unclear. A recent study reported morphological deformations in FSHD patient–derived myotubes in vitro (40, 41). To investigate the effect of iron deficiency or supplementation on myotube formation in vitro, we evaluated the morphology of DUX4-expressing myotubes treated with or without the iron chelator DFO or the iron donor ferrous ammonium sulfate (FAS) in vitro (Figure 8F). Although the fusion ability of multinucleated myotubes was unchanged among the groups, treatment with FAS, but not DFO, remarkably inhibited DUX4-induced deformation of myotubes (Figure 8, G–I). We measured the intracellular iron granularity in cultured myotubes with FerroOrange staining. Iron granules were accumulated in DUX4-Tg myotubes, which were not suppressed by FAS treatment (Supplemental Figure 7, A–D). Treatment with DFO did not improve malformation but reduced the iron granularity (Supplemental Figure 7, A–D). We also quantified the expression of iron metabolism–related proteins in DUX4-Tg myotubes under the DFO- and FAS-treated conditions. Unlike the results of the in vivo experiments (Figure 8, B and C), expression of IRP2, TFR, FTH, and FTL proteins was unaltered by DUX4 expression in myotubes (Supplemental Figure 7, E and F), even though the iron levels were increased.

To further determine the distribution of intracellular iron in myotubes, we performed Mito-FerroGreen staining to visualize the mitochondrial Fe^2+^ in living DUX4-Tg myotubes and found that the levels of mitochondrial Fe^2+^ were comparable between control and DUX4-Tg myotubes (Supplemental Figure 8, A and B). Lysosomes are master regulators of iron homeostasis and control the ferroptosis pathway (42). Costaining of FerroOrange (Fe^2+^) with LysoPrime Green (lysosomes) revealed that approximately 70% of the iron aggregates were identically localized to lysosomes in living DUX4-Tg myotubes (Supplemental Figure 8C). We also performed this costaining for isolated myofibers, but all myofibers were hypercontracted (dead) during the staining, indicating that the staining was not applicable for living myofibers. We observed increased levels of MitoSOX Red (mitochondrial superoxide) and BODIPY C11 (lipid peroxidation) fluorescence intensity in DUX4-Tg myotubes, which were suppressed by treatment with DFO or FAS (Supplemental Figure 9, A–C).

Overall, these results indicate that iron supplementation exerts a preventive effect on DUX4-induced muscle damage, both in vivo and in vitro, which is probably, in part, through the suppression of the ferroptosis-related pathway, but the expression dynamics of iron metabolism–related proteins in vitro did not entirely correspond with the data from the in vivo experiments.

### Iron supplementation alleviated retinal vascular abnormalities in DUX4-Tg mice.

**Figure 9:**
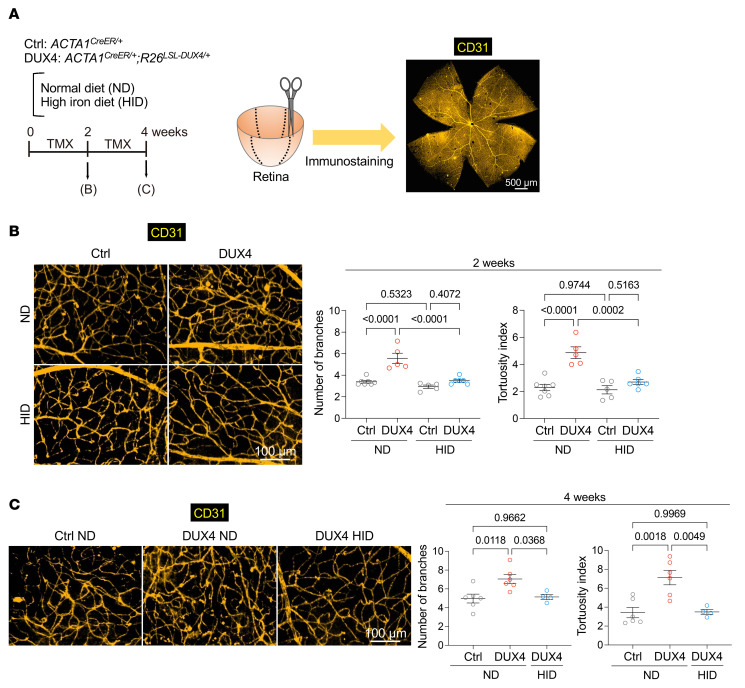
Iron supplementation improves retinal capillary abnormalities. (**A**) Time course. Control and DUX4-Tg mice were fed ND or HID mixed with TMX at a concentration of 0.03 mg/g feed for 2 or 4 weeks. Scale bar: 500 μm. (**B** and **C**) Retinas were isolated from control or DUX4-Tg mice and immunostained for CD31 to visualize blood vessels. Tortuosity index and number of blanches at 2 (*n* = 5–7) and 4 (*n* = 4–6) weeks. Scale bars: 100 μm. Data represent the mean ± SEM. *P* values were determined using 2-way or 1-way ANOVA followed by Tukey’s multiple-comparison post hoc test.

More than 50% of patients with FSHD exhibit retinal vasculopathy, which is a subclinical hallmark of FSHD (43). The severity of retinal tortuosity and the residual D4Z4 repeat array size are negatively correlated (44). In addition, retinal morphometric abnormalities, such as vessel branching, were reported in mice in which a doxycycline-inducible transgene encoding DUX4 and 3′ genomic DNA were introduced into a euchromatic region of the mouse X chromosome, where DUX4 is detected in retina (45). However, retinal vascular abnormalities have not yet been characterized in muscle-specific DUX4-expressing mice. We found that forced expression of DUX4 in the muscles resulted in an increase in the number of branches and tortuosity of the retinal capillaries (Figure 9, A–C), suggesting that retinal abnormalities are provoked by muscle-specific expression of DUX4. These abnormal capillaries became detectable in the second week following DUX4 induction prior to a reduction in grip strength, which was successfully prevented following iron supplementation (Figure 9, B and C).

### Ferroptosis compound library screening uncovered drugs to attenuate DUX4 toxicity.

**Figure 10:**
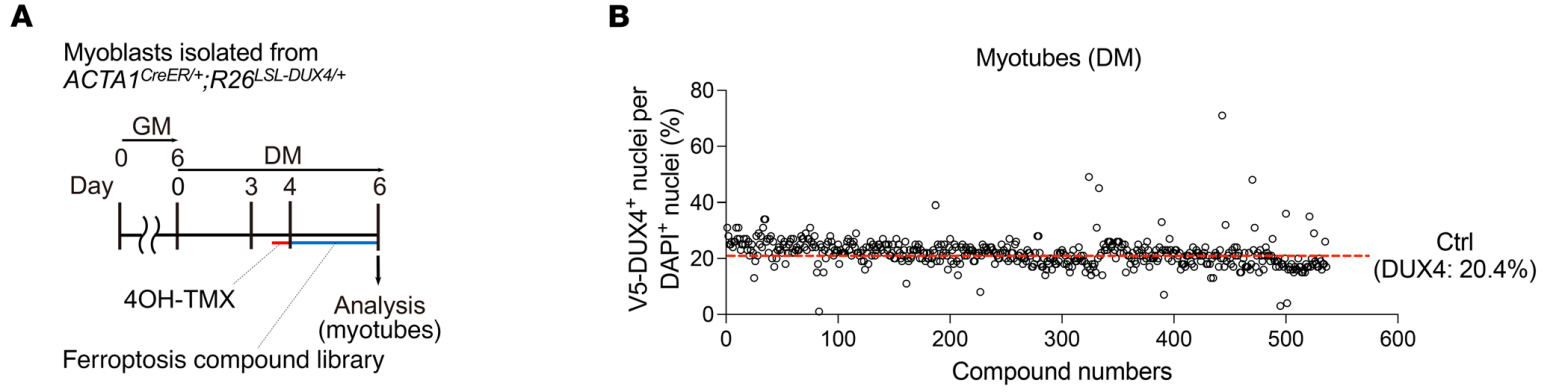
Ferroptosis compound library screening. (**A**) Evaluation of a compound library using myotubes expressing DUX4. Cultured myotubes were treated with the Ferroptosis Compound Library for 2 days in DM (*n* = 3). (**B**) Cell viability was evaluated by the rate of V5-DUX4 positivity. The decision for a hit compound was determined as ≥3 SD above the mean value of the control compound (20.4%). Eighteen hit compound targets are listed in Table 1.

For clinical implications, we sought to identify drug candidates for FSHD via a high-throughput screening assay focusing on the ferroptosis-related pathway, using a ferroptosis compound library that contained 536 compounds as inhibitors or activators related to ROS metabolism, iron metabolism, and ferroptosis signaling pathways. We observed that DUX4 expression was induced in myotubes differentiated from *ACTA1^CreER/+^ R26^LSL-DUX4+^* mouse–derived myoblasts after treatment with 4-hydroxytamoxifen and then cultured with the compounds for 2 days (Figure 10A). The DUX4 cytotoxicity was evaluated as cell viability using the ratio of V5-DUX4^+^ nuclei to total DAPI^+^ nuclei in a set of 3 independent experiments. The ratio of V5^+^ nuclei to total DAPI^+^ nuclei in the control group was 20.4% (Figure 10B). A hit compound was determined as ≥3 SD above the mean value of the control compound, according to a previous study (46). High-throughput screening identified compounds that attenuated DUX4 cytotoxicity; however, we excluded contaminant compounds, including RSL3 and oxfendazole, from the hit compounds that are known to exert cytotoxicity. We identified 18 potential compounds for drug development (Table 1). As expected, the antioxidant tempol (14) and the steroidal estrogen quinestrol (47), but not iron chelators, were found among the hit compounds.

### Ferrostatin-1 alleviated physical function in DUX4-Tg mice.

**Figure 11:**
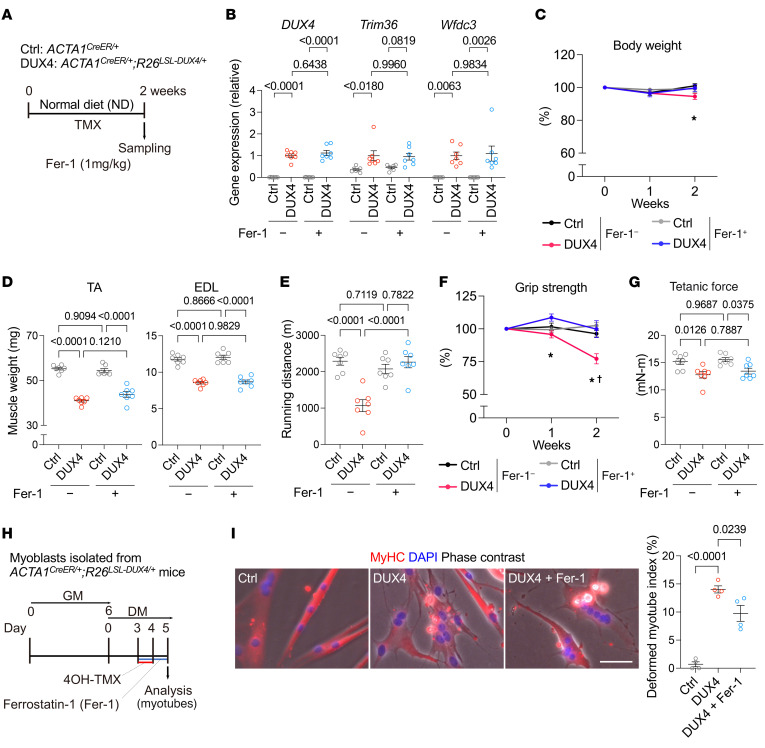
Improvement of pathologies by ferrostatin-1 in DUX4-Tg mice. (**A**) Time course. Ferrostatin-1 (Fer-1; 1 mg/kg body weight) was intraperitoneally injected every day. (**B**) qPCR analysis of *DUX4* and its target genes in quadriceps muscle (*n* = 7). (**C**) Body weights (*n* = 7). *P < 0.05, control saline vs. DUX4 saline. (**D**) Muscle weights (*n* = 7). (**E**) Running test (*n* = 7). (**F**) Grip strength (*n* = 7). *P < 0.05, DUX4 saline vs. DUX4 Fer-1; ^†^*P* < 0.05, control saline vs. DUX4 saline. (**G**) Tetanic muscle force (*n* = 7). (**H**) Cultured myotubes were treated with 5 μM Fer-1 for 48 hours in DM. (**I**) Morphological analysis determined deformed myotube index (*n* = 4). Scale bar: 100 μm. Data represent the mean ± SEM. *P* values were determined using 1-way or 2-way ANOVA followed by Tukey’s multiple-comparison post hoc test.

We identified ferrostatin-1 (Fer-1), a potent inhibitor of lipid peroxidation (33), as the most effective compound for improving cell viability against DUX4 cytotoxicity using compound library screening (Table 1). To strengthen the evidence that the ferroptosis-related pathway could be a therapeutic target for FSHD, we tested the effect of Fer-1 on DUX4-Tg mice in vivo (Figure 11A). Treatment with Fer-1 for 2 weeks in DUX4-Tg mice in vivo remarkably improved grip strength and running performance without affecting gene expression profiles, muscle weight, and muscle force generation (Figure 11, B–G), consistent with the HID-fed (Figure 5, E and F) and FCM-treated (Figure 6, I and K) conditions. We also confirmed that Fer-1 administration prevented the DUX4-induced deformed myotube formation (Figure 11, H and I).

## Discussion

In the present study, we described an abnormal accumulation of iron in the muscles of patients with FSHD, especially in those with a lower number of D4Z4 repeats. We also observed excessive iron deposition in the myofibers of DUX4-Tg mice in vivo and in DUX4-expressing myotubes in vitro. According to the previous studies on the beneficial effects of iron chelators on sarcopenia and DMD (17, 18, 20, 48), we predicted that reducing iron levels would improve FSHD pathologies. However, iron insufficiency did not improve any of the effects on physical functions but rather promoted the reduction of muscle strength in DUX4-Tg mice. Surprisingly, iron supplementation markedly ameliorated voluntary locomotor activity, treadmill running ability, and grip strength in DUX4-Tg mice. These unexpected results provide evidence that DUX4 toxicity is attenuated by iron supplementation in mice in vivo.

High-throughput inhibitor screening performed by Bosnakovski et al. revealed that most compounds protecting against DUX4 toxicity were antioxidant associated, suggesting that oxidative stress is a major downstream pathway of DUX4 (46). No compounds associated with caspase activation–induced cell death were found during the screening (46). In the present study, we focused on ferroptosis, a recently discovered iron-dependent cell death pathway (33, 49). We demonstrated that the ferroptosis-related pathway was altered in DUX4-Tg muscles, which was suppressed by iron supplementation. The ferroptosis pathway involved in muscle is not well characterized. GPX4 is a major antioxidant enzyme that prevents ferroptosis (39). Muscle-specific GPX4 deletion in mice results in activation of the ferroptosis pathway and muscle atrophy (50). Intriguingly, this GPX4 inactivation–induced muscle atrophy is mediated in a lysosome-dependent but proteasome-independent manner (50). Lysosomes are master regulators of iron turnover and controls the ferroptosis pathway (42). We found that iron-dense granules were mainly localized to lysosomes in DUX4-Tg myotubes. Moreover, our transcriptome analysis showed that iron supplementation attenuated the DUX4-induced upregulation of lysosomal genes, indicating that iron supplementation influenced lysosomal activity and the ferroptosis-related pathway in DUX4-Tg muscles.

Treatment with the iron donor FAS prevented morphological deformation and reduced the levels of mitochondrial ROS and lipid peroxidation in DUX4-expressing myotubes in vitro. Indeed, the supplementary iron–induced reduction of DUX4 toxicity could, at least in part, be mediated through the suppression of lipid peroxidation in the muscle. In support of this interpretation, our high-throughput compound screening of the ferroptosis pathway identified Fer-1, a potent inhibitor of lipid peroxidation, and treatment with Fer-1 in vivo remarkably improved physical function in DUX4-Tg mice. Lipid peroxidation is a devastating reaction that occurs in the plasma membrane and facilitates cell death through ferroptosis (51). Oxidative stress–induced deficits in plasma membrane repair have been observed in DUX4-expressing myofibers and may be involved in the pathogenesis of FSHD (52). Therefore, we assume that supplementary iron attenuates the disruption of the plasma membrane and consequent muscle damage in DUX4-Tg muscle by inhibiting lipid peroxidation.

More than half of patients with FSHD exhibit retinal symptoms as subclinical hallmarks (27, 43). One of the most striking findings of this study was that muscle-specific DUX4-Tg mice exhibited retinal vascular abnormalities and that iron supplementation improved not only muscle pathologies but also retinal abnormalities. We predicted that aberrant regulation of muscle-derived factors, such as myokines or exosomes, might be involved in the pathogenesis of the retina in DUX4-Tg mice. Therefore, it is crucial to elucidate organ-organ interactions in the pathogenesis of FSHD.

Although excess iron is known to trigger ferroptosis, our results revealed an opposing effect: iron supplementation suppressed ferroptosis-related pathways and ameliorated the pathology in DUX4-Tg mice in vivo. In the present study, we observed elevated iron levels in both the serum and muscle of DUX4-Tg mice, which may correspond to the increased plasma ferritin levels reported in patients with FSHD (53). Notably, iron supplementation through HID feeding or FCM treatment led to iron accumulation in the liver, a primary iron storage organ, but paradoxically decreased iron levels in the muscle of DUX4-Tg mice. This underlying mechanism remains to be elucidated; however, we speculate that a negative-feedback response induced by supplemental iron may prevent excessive iron accumulation in muscle tissue. Consequently, the observed amelioration of pathology may be attributed to reduced iron uptake into DUX4-Tg muscle (e.g., via downregulation of TFR expression), thereby indirectly suppressing ferroptosis-related pathways. It is also possible that the beneficial effects of iron supplementation in DUX4-Tg mice involve additional mechanisms beyond the attenuation of ferroptosis.

In summary, we demonstrated that DUX4 induces abnormal iron metabolism in muscles, providing a better understanding of the pathophysiology of DUX4-provoked toxicity. However, further investigation is required to understand the molecular mechanisms by which iron supplementation or Fer-1 treatment improves physical function in DUX4-Tg mice, particularly how the iron metabolism and ferroptosis-related pathways are regulated at both intra- and intercellular levels. Our findings indicate that iron supplementation is a promising and readily available therapeutic option for the treatment of FSHD.

## Methods

### Sex as a biological variable.

This study examined male and female animals, and similar findings are reported for both sexes. Sex of the human samples was not disclosed.

### Human samples and iron histochemistry.

Patients with FSHD1 were divided into 2 groups: one with 1–3 D4Z4 unit repeats and the other with 4–5 D4Z4 unit repeats. Samples from individuals with more than 13 D4Z4 repeats and some medical symptoms but no obvious pathologies in the muscle were used as controls.

Iron histochemistry of human muscle biopsy samples was performed as previously described (54). Human muscle tissues were sliced into 10-μm-thick sections using a cryostat (Leica Biosystems), fixed in a 4% paraformaldehyde solution in phosphate-buffered saline (PFA/PBS) for 5 minutes, washed with distilled water, and incubated with 7% potassium ferricyanide in a 3% HCl solution at 37°C for 1 hour. Subsequently, tissue sections were rewashed with distilled water and incubated with 0.75 mg/mL 3,3′-diaminobenzidine and 0.015% H_2_O_2_ for 30 minutes at room temperature. After washing with distilled water, sections were air-dried before mounting. The intensity of iron staining in the sections was quantified using ImageJ (NIH) from digital images captured with a DP80 camera (Olympus).

### Animals.

Animals were housed in a pathogen-free environment. All animals were housed under a 12-hour dark/12-hour light cycle (light from 0700 to 1900 hours) at 22°C ± 1°C with ad libitum food and water. *ACTA1^CreER/+^* mice (24) (stock 031934) and *R26^LSL-DUX4^* mice (25) (stock 032779) were obtained from The Jackson Laboratory Japan. *R26^LSL-DUX4/+^* mice were crossed with *ACTA1^CreER/+^* mice to generate *ACTA1^CreER/+^ R26^LSL-DUX4/+^* mice. *ACTA1^CreER/+^ R26^LSL-DUX4/+^* mice were crossed with *Irp2^–/–^* mice (29, 30) to generate *ACTA1^CreER/+^ R26^LSL-DUX4/+^* *Irp2^–/–^* mice.

For the injection protocol, TMX (Sigma-Aldrich) dissolved in corn oil was intraperitoneally administered (5 mg/kg body weight) 3 times per week for 2 weeks. For the feeding protocol, TMX was mixed with ND (320 ppm iron, CE-2, CLEA), ND2 (50 ppm iron, TD.160777, ENVIGO), IDD (2–6 ppm iron, TD.80396, ENVIGO), or HID (20,000 ppm iron, TD.10066, ENVIGO) at a concentration of 0.03 mg/g feed (55). DFO (D9533, Sigma-Aldrich) dissolved in PBS was intraperitoneally injected (300 mg/kg body weight). DFX (HY-17359, MedChemExpress) dissolved in corn oil was administered via oral gavage at a dose of 20 mg/kg body weight. FCM (Vifor Pharma) dissolved in saline was injected into the tail vein at a dose of 15 mg/kg body weight. Ferrostatin-1 (S7243, Selleck Biotech) dissolved in saline containing 2% DMSO was intraperitoneally injected (1 mg/kg body weight). Appropriate vehicle controls were used for each treatment condition. Biochemical parameters of mouse serum were measured using BioMajesty (JCA-BM6050) at the Institute of Resource Development and Analysis, Kumamoto University.

All experiments used male mice except for those shown in Figure 3 and Supplemental Figure 3, which used female mice, and Supplemental Figure 4, which used both male and female mice. All experiments were performed using 9- to 23-week-old mice.

### Grip strength and tetanic muscle force.

Whole-limb grip strength was measured using a grip strength meter (Columbus Instruments). Peak tension (in newtons) was recorded when the mouse released its grip. Two sets of 10 successive measurements were performed for each mouse, and the maximal strength was used for data analysis.

Tetanic muscle force was measured in the tibialis anterior (TA) muscle using the Whole Animal Muscle Test System (Aurora Scientific) as previously described (56). Briefly, mice were anesthetized with isoflurane and placed on a 37°C warming plate throughout the procedure. The right foot was fixed to the footplate connected to the servomotor, and the knee was immobilized. The fixed lower leg was shaved to locate the TA muscle and subcutaneously stimulated with 2 needle electrodes at 5 mA. Tetanic contractions were elicited by stimulation for 350 milliseconds at a frequency of 100 Hz, and the maximal force was determined.

### Rotarod test.

Motor coordination and fatigue tolerance were determined using the rotarod test (BioResearch Center). The rotarod program, starting at 6 rpm, was evaluated using a constant-speed protocol. The acceleration protocol was initiated at 4 rpm and increased by 1 rpm every 8 seconds to 40 rpm for up to 300 seconds. The maximum values of 3 measurements were used.

### Voluntary locomotor activity.

Each mouse was individually housed in a cage, and voluntary locomotor activity was evaluated every 10 minutes using SUPERMEX (Muromachi Kikai). The mice were housed under a 12-hour dark/12-hour light cycle with ad libitum access to food and water. After acclimatization, activity was measured over a 24-hour period. The data were shown as 24-hour or 12-hour activity.

### Antibodies.

The primary antibodies used were as follows: Rat anti–laminin α-2 (sc-59854, 4H8-2; 1:800 for immunofluorescence [IF]), mouse anti-ACSL4 (sc-365230, F-4; 1:5,000 for Western blot [WB]), and mouse anti–8-OHdG (sc-66036, 15A-3; 1:1,000 for IF) antibodies were purchased from Santa Cruz Biotechnology. Mouse anti–myosin heavy chain (anti-MyHC) antibody (MF20; 1:5 for IF) was obtained from the Developmental Studies Hybridoma Bank. Rat anti-CD31 antibody (102408, 390; 1:400 for IF) was purchased from BD Biosciences. Mouse anti–4-HNE antibody (MAB3249, 198960; 1:5,000 for WB) was obtained from R&D Systems. Mouse anti-IRP2 antibody (MABS2030-100UG, 3B11; 1:1,000 for WB) was purchased from Sigma-Aldrich. Mouse anti-TFR antibody (13-6800, H68.4; 1:5,000 for WB) and HRP-conjugated mouse anti-V5 antibody (R961-25; 1:2,000 for WB) were purchased from Thermo Fisher Scientific. Rabbit anti-SLC40A1 antibody (NBP1-21502; 1:5,000 for WB) was obtained from Novus Bio. Rabbit anti-FTL (ab69090; 1:5,000 for WB), rabbit anti-FTH (ab65080; 1:5,000 for WB), and rabbit anti-GPX4 (ab125066, EPNCIR144; 1:5,000 for WB) antibodies were purchased from Abcam.

We purchased the following secondary antibodies from Thermo Fisher Scientific: Alexa Fluor 555–conjugated goat anti-mouse IgG (A-21422; 1:800 for IF), Alexa Fluor 546–conjugated goat anti-rabbit IgG (A-11035; 1:800 for IF), Alexa Fluor 488–conjugated goat anti-rabbit IgG (A-11034; 1:800 for IF), Alexa Fluor 488–conjugated goat anti-mouse IgG (A-32723; 1:800 for IF), Alexa Fluor 546–conjugated goat anti-rat IgG (A-11081; 1:800 for IF), and Alexa Fluor 488–conjugated goat anti-rat IgG (A-11006; 1:800 for IF). We purchased HRP-conjugated anti-rabbit IgG (7074; 1:5,000 for WB) and HRP-conjugated anti-mouse IgG (7076; 1:5,000 for WB) antibodies from Cell Signaling.

### Immunofluorescence and imaging.

Immunohistochemical analysis was performed, as previously described (57). We isolated TA muscles from mice, immediately froze them in 2-methylbutane cooled with liquid nitrogen, and stored them at –80°C until analysis. Tissues were sliced into 10-μm-thick sections using a cryostat (Leica Biosystems).

Retinas were isolated from the eyeballs of the mice after first fixation with 4% PFA/PBS on ice for 30 minutes. Retinas were then fixed with 4% PFA/PBS at 4°C overnight, after the second fixation in microwave on ice for 15 seconds. Samples were incubated with primary antibodies at 4°C overnight, following 0.1% Triton X/1% bovine serum albumin/PBS at room temperature for 1 hour after washing 3 times with 0.1% Triton X/PBS. The samples were quantified using methods modified from previous studies (58, 59). The branches were measured as the number of inflection points on the straight-line distance between the end points (300 μm). The tortuosity index was calculated as the total distance multiplied by the number of curves on the straight-line distance between the endpoints (300 μm) divided by 300 μm. Two *Z*-stack images per sample were used, and 3 fields on each *Z*-stack image were analyzed (12 fields per sample).

To visualize Fe^2+^, cultured myotubes and freshly isolated myofibers were costained with FerroOrange (F374, Dojindo) and Hoechst 33342 according to the manufacturer’s instructions. Fe^2+^-dense granules accumulated in the cytoplasm or around the nucleus of myofibers were measured. Briefly, the average value of Fe^2+^-dense granules was quantified at 3 focal points of randomly selected locations using *Z*-stacks. The accumulation of Fe^2+^-dense granules in the cytoplasm of myotubes was measured. Briefly, the average value of the number of Fe^2+^-dense granules was quantified using 10–20 myotubes per sample.

To visualize mitochondrial Fe^2+^, living cultured myotubes were costained with Mito-FerroGreen (M489, Dojindo) and Hoechst 33342 according to the manufacturer’s instruction. To evaluate lipid peroxidation, living cultured myotubes were costained with BODIPY 581/591 C11 (D3861, Thermo Fisher Scientific) and Hoechst 33342 according to the manufacturer’s instruction. The lipid peroxidation levels were quantified as the ratio of green fluorescence (oxidized form) to red fluorescence (reduced form). To quantify mitochondrial superoxide levels, living cultured myotubes were costained with MitoSOX (M36008, Thermo Fisher Scientific) and Hoechst 33342 in accordance with the manufacturer’s instructions. The fluorescence intensities were measured using CX5 (Thermo Fisher Scientific).

To visualize the colocalization of Fe^2+^ granules and lysosome, living cultured myotubes were costained with FerroOrange, LysoPrime Green (L261, Dojindo), and Hoechst 33342 in accordance with the manufacturer’s instructions. The area of LysoPrime Green^+^ per FerroOrange^+^ was calculated using the colocalization function of cellSens (Olympus).

The samples were visualized using Alexa Fluor–conjugated secondary antibodies and viewed under a fluorescence microscope (IX83, Olympus). Digital images were acquired using a DP80 camera with cellSens software (Olympus) or an all-in-one microscope (BZ-X710, KEYENCE). Representative images of the retinas were obtained using a confocal microscope (BC43, Oxford Instruments).

### Immunoblotting.

Protein lysates were obtained from homogenized quadriceps muscle tissues using radioimmunoprecipitation assay (RIPA) buffer (FUJIFILM-Wako). The protein concentration was quantified using the Pierce BCA Protein Assay Kit (Thermo Fisher Scientific). Primary antibodies were diluted in 5% skim milk and incubated with membranes containing electrophoretically transferred proteins at 4°C overnight. The membranes were washed 3 times with PBST and incubated with secondary antibodies diluted in 5% skim milk at room temperature for 1 hour. Secondary antibodies were visualized by measurement of chemiluminescence using an LAS-4000 digital luminescent image analyzer (GE Healthcare). Ponceau staining (P7170, Sigma-Aldrich) was used as an internal control for normalization.

### Quantitative real-time PCR analysis.

Total RNA was extracted from muscle tissues using ISOGEN II (Nippon Gene) or the RNeasy kit (QIAGEN), according to the manufacturer’s instructions. cDNA was prepared using a ReverTra Ace kit with genomic DNA remover (TOYOBO), and qPCR was performed using THUNDERBIRD STBR mix (TOYOBO) and a CFX96 Touch Deep Well Real-Time PCR Detection System (Bio-Rad). The primers used were as follows: DUX4, 5′-CAGGCGCAACCTCTCCTAGA-3′ (forward) and 5′-GCCCGGTATTCTTCCTCGCT-3′ (reverse); Trim36, 5′-TGAAAGTGGGAGTTGCTTCC-3′ (forward) and 5′-GAATCAAAACAGGCGTCCTC-3′ (reverse); Wfdc3, 5′-CTTCCATGTCAGGAGCTGTG-3′ (forward) and 5′-ACCAGGATTCTGGGACATTG-3′ (reverse); TATA box–binding protein (TBP), 5′-CAGATGTGCGTCAGGCGTTC-3′ (forward) and 5′-TAGTGATGCTGGGCACTGCG-3′ (reverse).

### Transcriptome analysis.

Total RNA was obtained from the gastrocnemius and plantaris muscles of ND- and HID-fed mice using ISOGEN II and the RNeasy kit. Library preparation and RNA-Seq were performed by Novogene (Beijing, China). The data were generated from approximately 60 million reads per sample using an Illumina NovaSeq platform with paired-end 150 bp sequencing strategies. The data were converted into FASTQ files and mapped to reference genomes and transcripts for *Mus musculus* mm10 (GENCODE vM23/Ensembl 98) using Strand NGS v3.4 software (Strand Life Sciences). The data were analyzed using RNAseqChef (60) to generate principal component analysis plots, heatmaps, and graphs categorized by gene expression. Multiple differentially expressed genes were identified by application of the following thresholds: fold change > 1.2, FDR < 0.05, and base mean = 0. Read counts were normalized using DEseq2.

### Cell culture and compound screening.

Primary myoblasts were isolated from the muscles using either the individual myofiber method (28) or the preplating method (61) and cultured in growth medium, as previously described. Myogenic differentiation from myoblasts to myotubes was induced in differentiation medium (DM; DMEM supplemented with 2% horse serum and 1% penicillin-streptomycin) for 3 days. After the differentiation, 1 μM 4-hydroxytamoxifen (4OH-TMX; Sigma-Aldrich) was added to DM for 24 hours to induce DUX4 expression, and differentiated myotubes were then analyzed 24 hours later. Myotube formation was defined as MyHC^+^ cells containing more than 4 DAPI^+^ nuclei. The fusion index was described as the ratio of the number of DAPI^+^ nuclei in the myotubes to the total number of MyHC^+^DAPI^+^ nuclei (62). The deformed myotube index was defined as the ratio of the number of myotubes containing more than 4 filopodia to the total number of MyHC^+^DAPI^+^ nuclei; DFO (ab120727, Abcam) and FAS (091-00855, FUJIFILM) were used as an iron chelator and iron donor, respectively. For compound screening, differentiated myotubes were treated with the Ferroptosis Compound Library (L6400, Selleck Biotech) using a benchtop multi-pipette (EDR-384SR, software v2.79, BIOTEC) in DM for 2 days, followed by treatment with 4OH-TMX for 12 hours in DM. All cultures were incubated at 37°C and 5% CO_2_.

### Measurement of iron contents in tissues and serum.

The iron contents in quadriceps muscle, liver, and serum were measured using a Metallo assay kit (FE31M, Metallogenics) according to the manufacturer’s instructions. Briefly, the muscle and liver were homogenized using RIPA buffer (FUJIFILM-Wako). Tissue and serum samples were mixed with the R-A Buffer (Metallogenics) for 5 minutes, and the baseline absorbance (OD1) was determined. Then, the R-R Chelate (Metallogenics) color was added to samples for 5 minutes, and the absorbance (OD2) was determined. Iron contents (OD2 – OD1) of muscle and liver were normalized to protein concentrations.

### Glutathione quantification.

Reduced glutathione (GSH) and oxidized glutathione (GSSG) were analyzed using a GSSG/GSH quantification kit (G257, Dojindo) according to the manufacturer’s instructions. Briefly, muscle tissue was frozen in liquid nitrogen and homogenized in 5% 5-sulfosalicylic acid dihydrate (190-04572, FUJIFILM Wako Pure Chemical Corp.), and the insoluble fraction was removed using centrifugation. The supernatant was collected and analyzed by measurement of the absorbance at OD 405 nm.

### Statistics.

Statistical analyses were performed using Prism version 10 (GraphPad Software Inc.). Student’s 2-tailed unpaired *t* test was used for statistical comparisons between 2 conditions. For comparisons of more than 2 groups, data were analyzed using 1-way or 2-way ANOVA, followed by Tukey’s post hoc multiple comparisons. All data represent the mean ± SEM. “NS” indicates results that are not statistically significant. A *P*-value of < 0.05 was considered significant.

### Study approval.

All patients provided informed consent for the use of their samples for research after diagnosis. This study was approved by the Ethics Committee of the National Center of Neurology and Psychiatry (A2019-123 and A2021-009). All animal experiments were approved by the Institutional Animal Care and Use Committee of Kumamoto University (A2022-075 and A2024-096).

### Data availability.

Raw RNA-Seq datasets are available from the Gene Expression Omnibus public depository under accession number GSE261617. All data used in the figures are reported in the Supporting Data Values file.

## Author contributions

KN conducted the experiments, interpreted the data, assembled the input data, and wrote the manuscript. HPOQ performed the animal experiments. NH performed the iron content measurement. SF performed the glutathione quantification. TM, KIN, and SH provided key materials. YS and IN performed experiments on human samples. YO designed the experiments, interpreted the data, assembled the input data, and wrote the manuscript. All the authors reviewed and approved the final manuscript.

## Supplementary Material

Supplemental data

Unedited blot and gel images

Supporting data values

## Figures and Tables

**Table 1:**
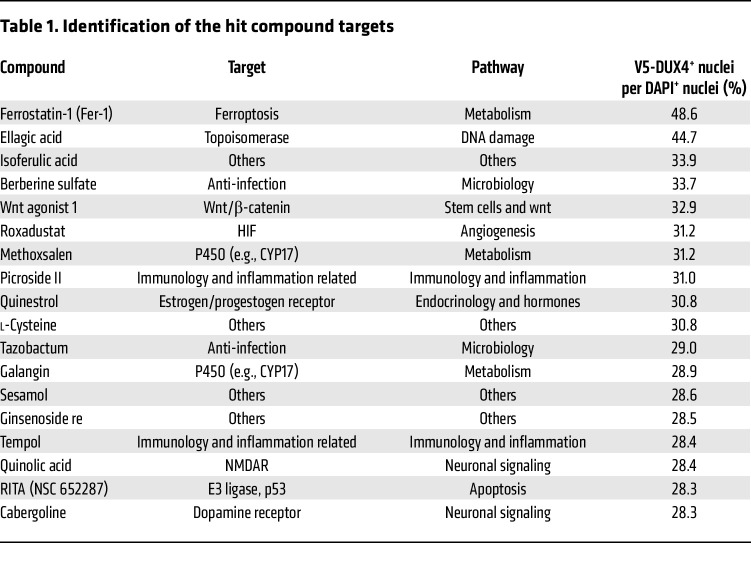
Identification of the hit compound targets

## Version 1 07/01/2025

In-Press Preview

## Version 2 09/02/2025

Electronic publication
