# Supplementary material for: Iron supplementation alleviates pathologies in a mouse model of facioscapulohumeral muscular dystrophy
Source: J Clin Invest. 2025 Jul 1;135(17):e181881. doi: 10.1172/JCI181881 (PMC12404756; doi:10.1172/JCI181881)

Supplementary Figure 1

A

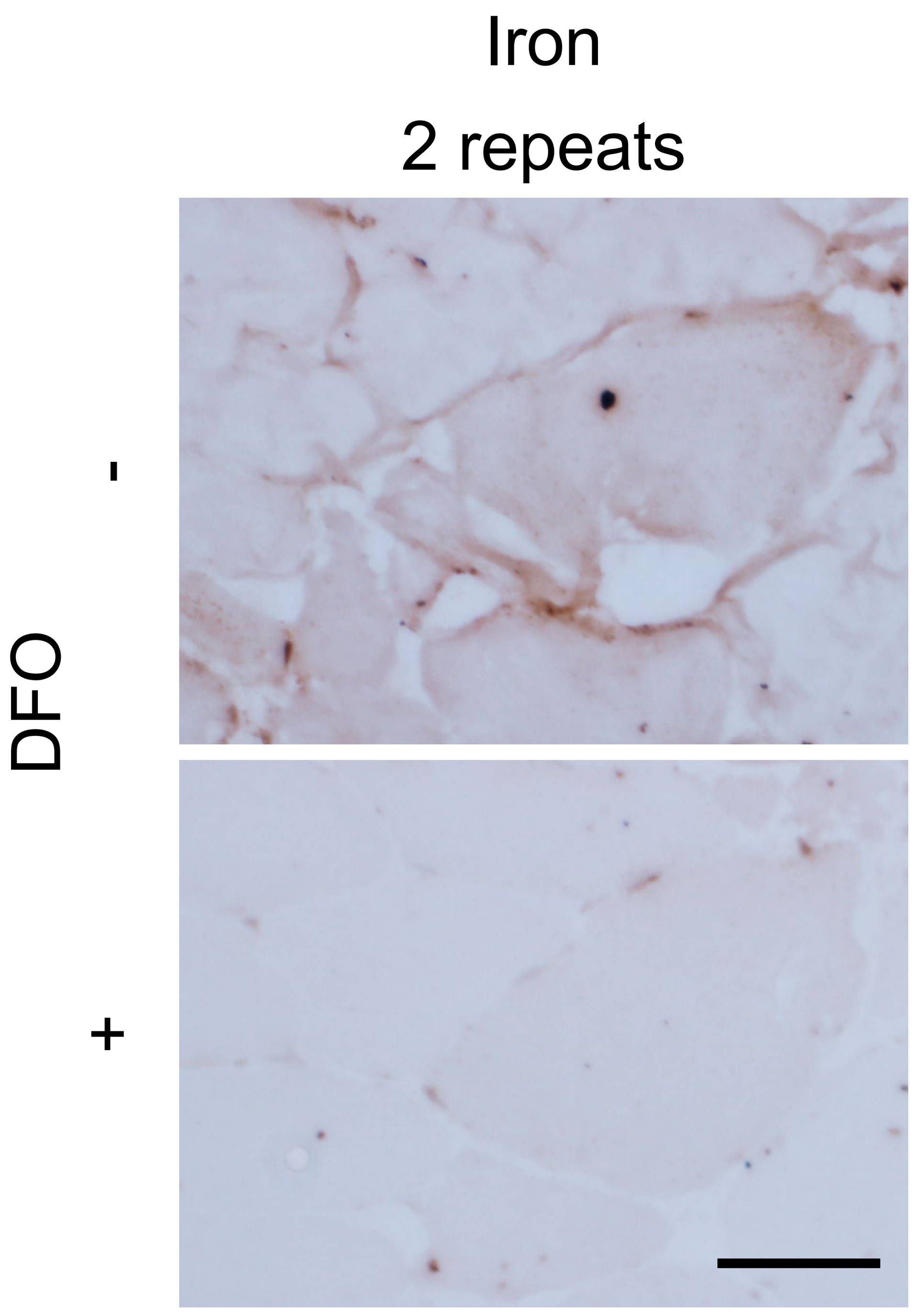

Supplementary Figure 2

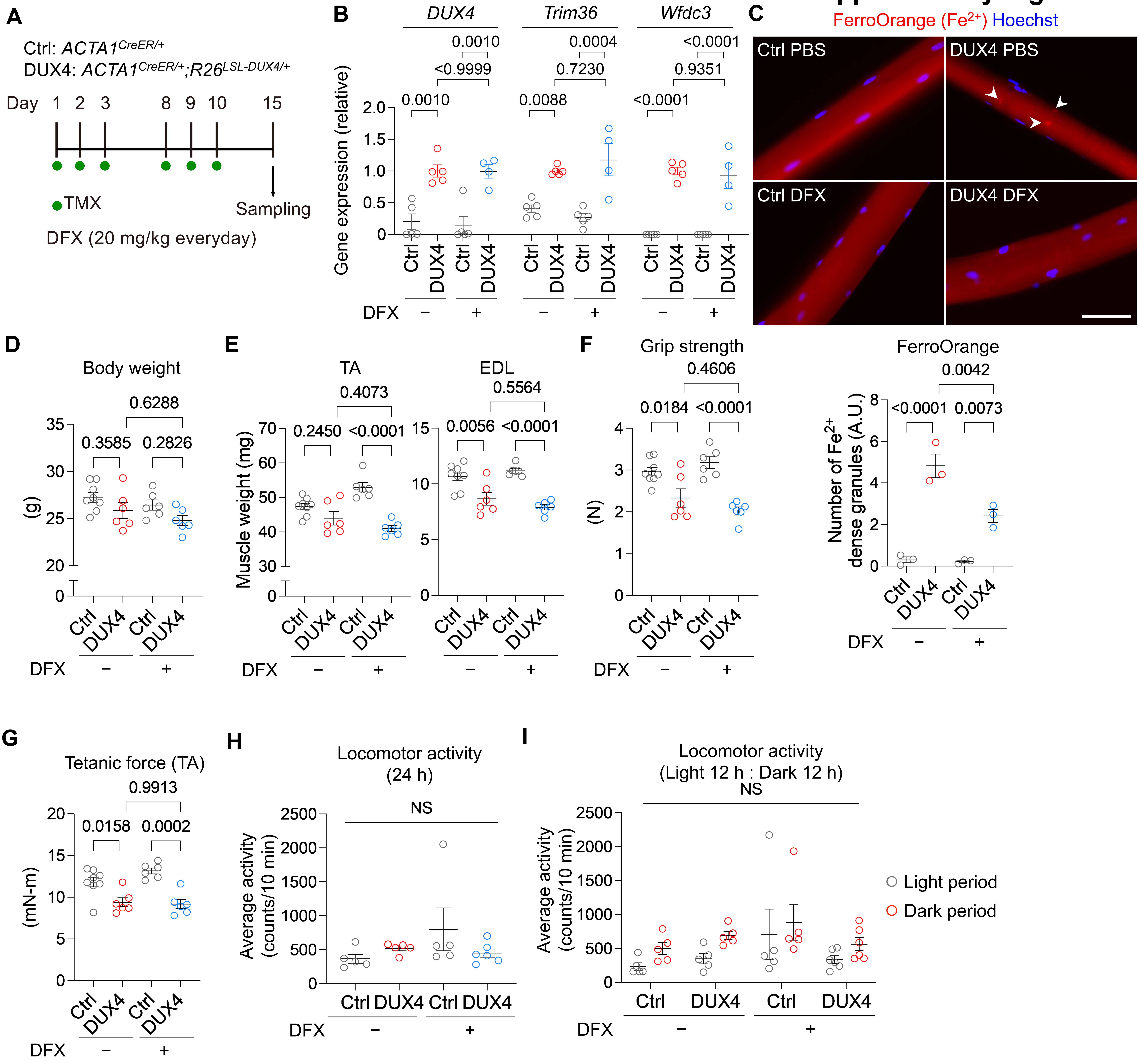

Supplementary Figure 3

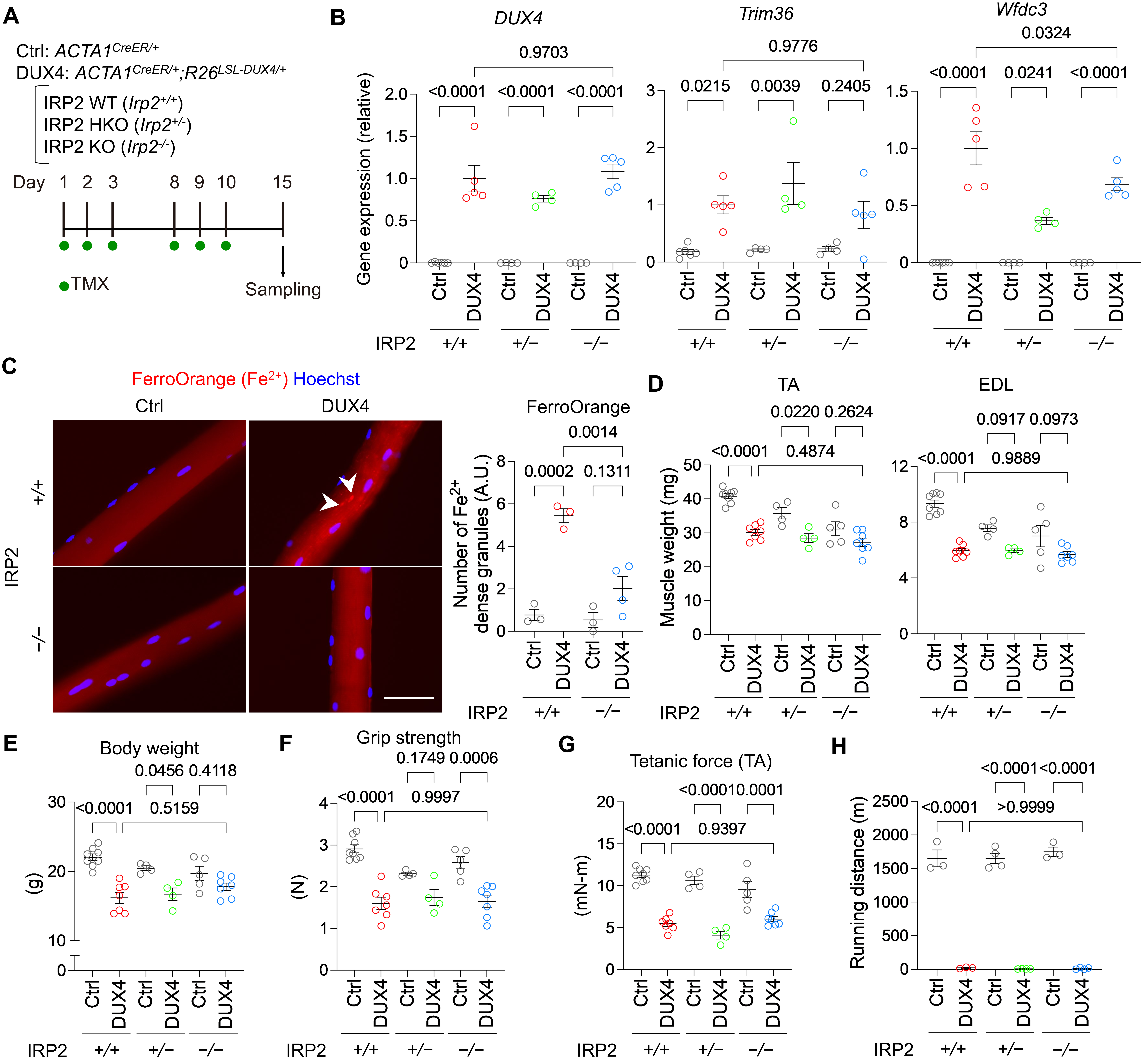

Supplementary Figure 4

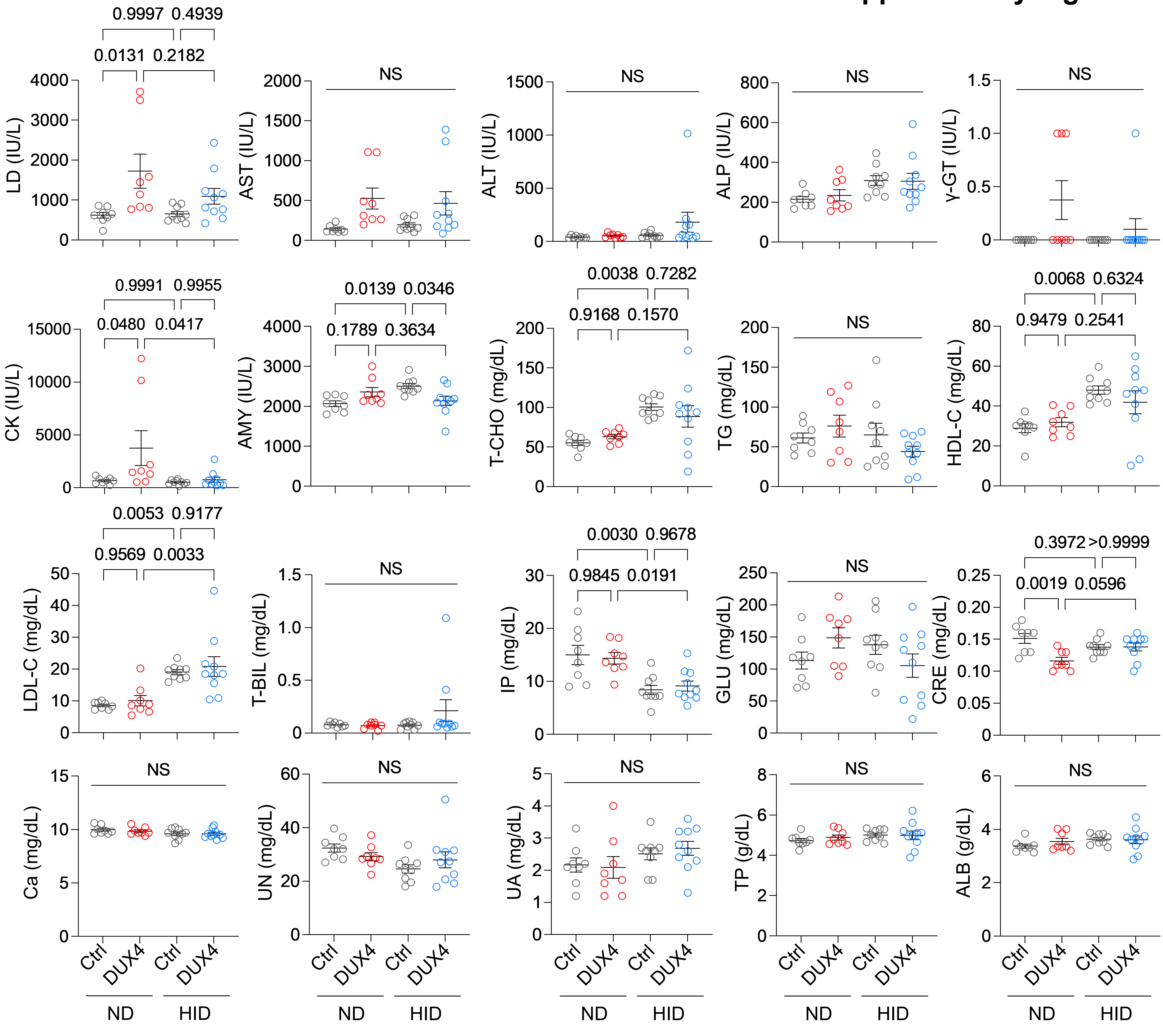

Supplementary Figure 5

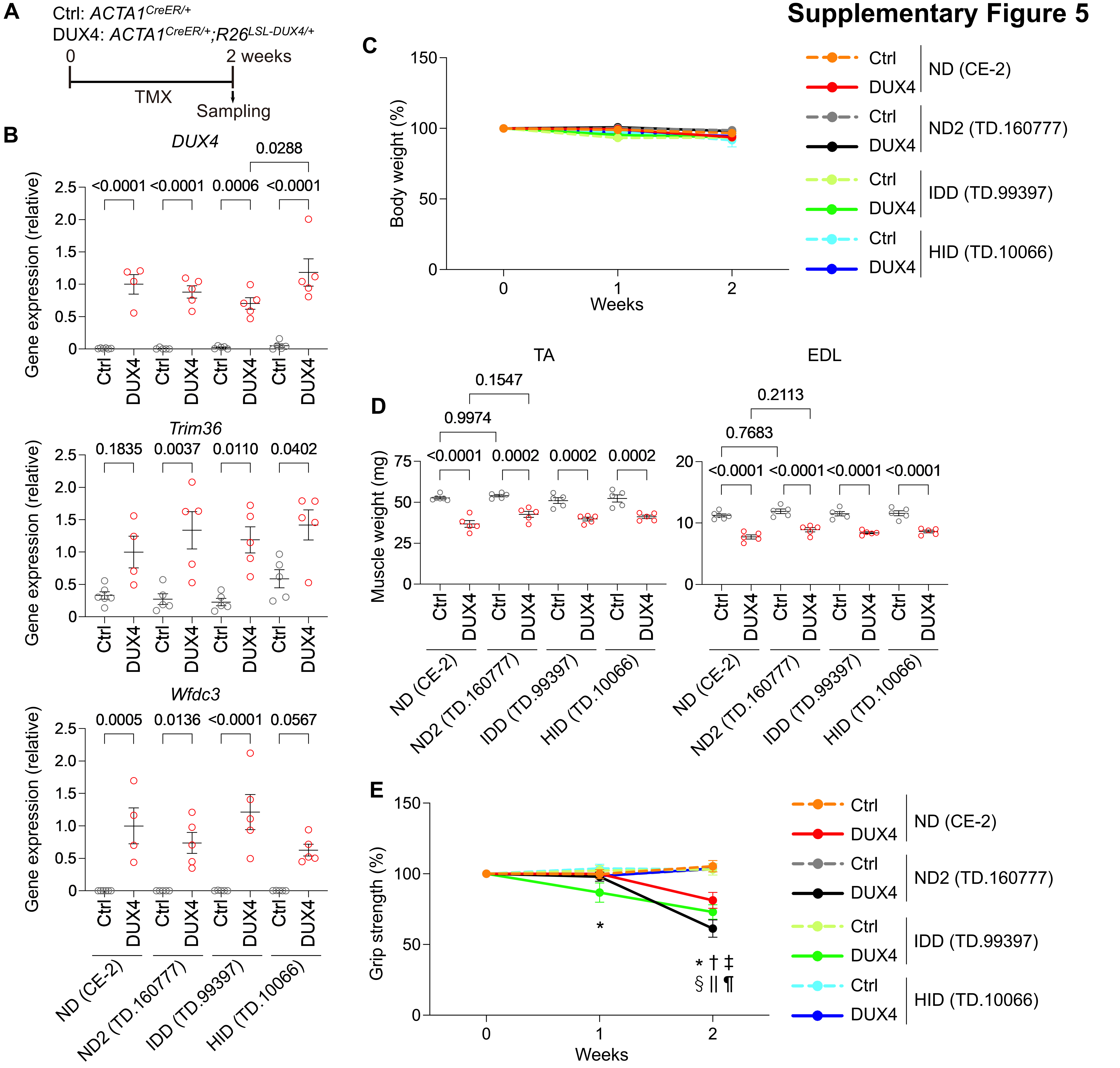

Supplementary Figure 6

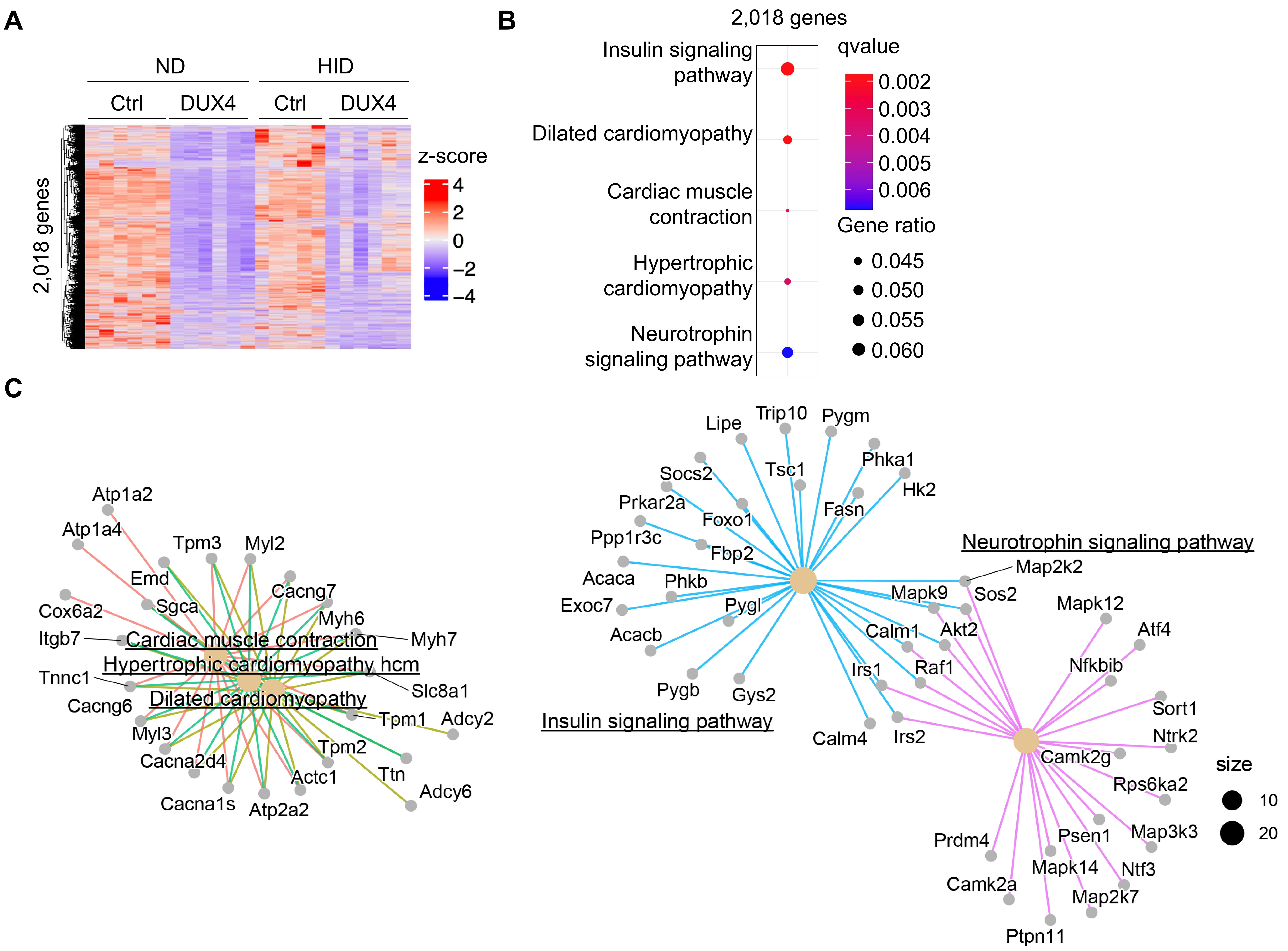

Supplementary Figure 7

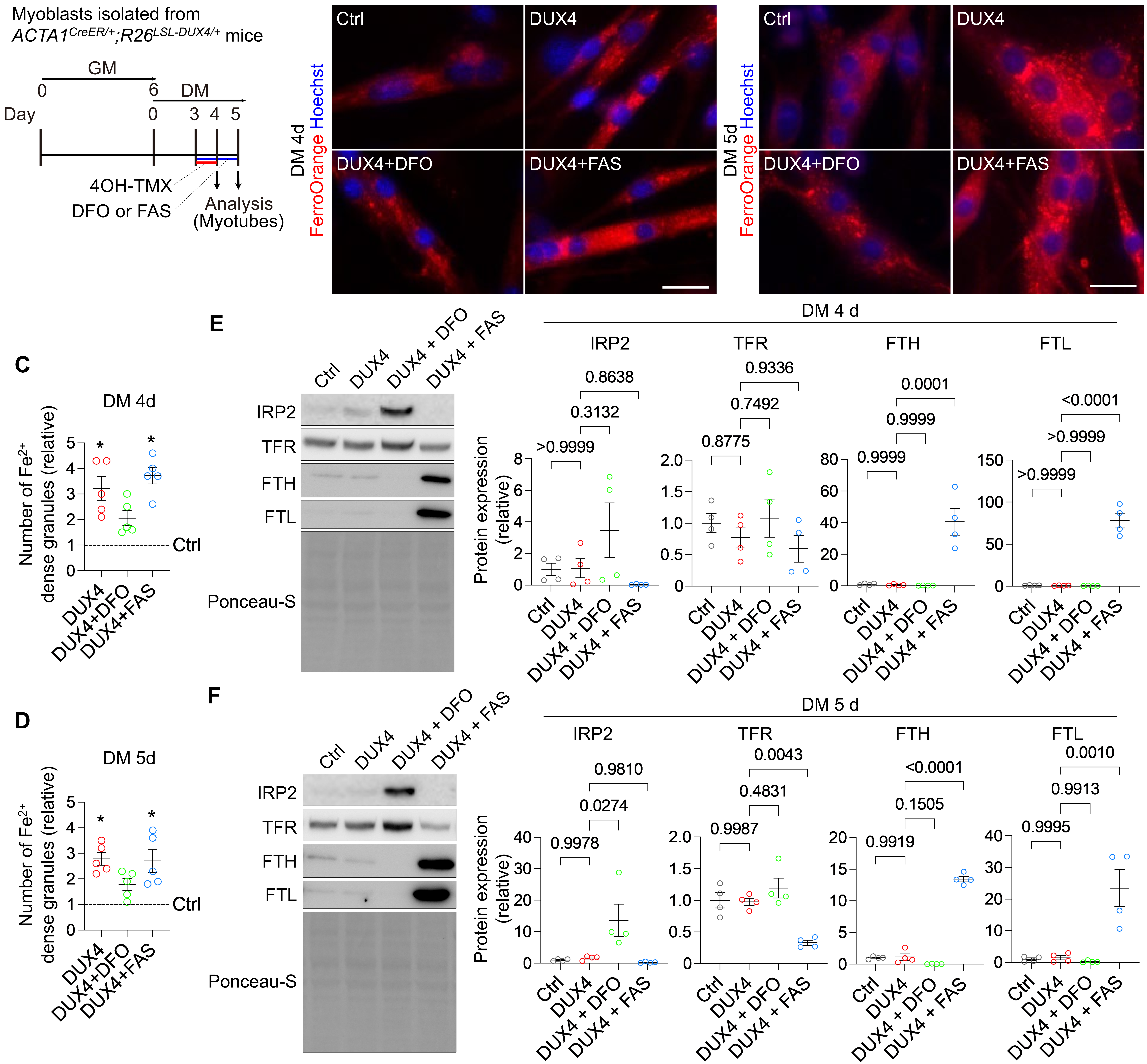

Supplementary Figure 8

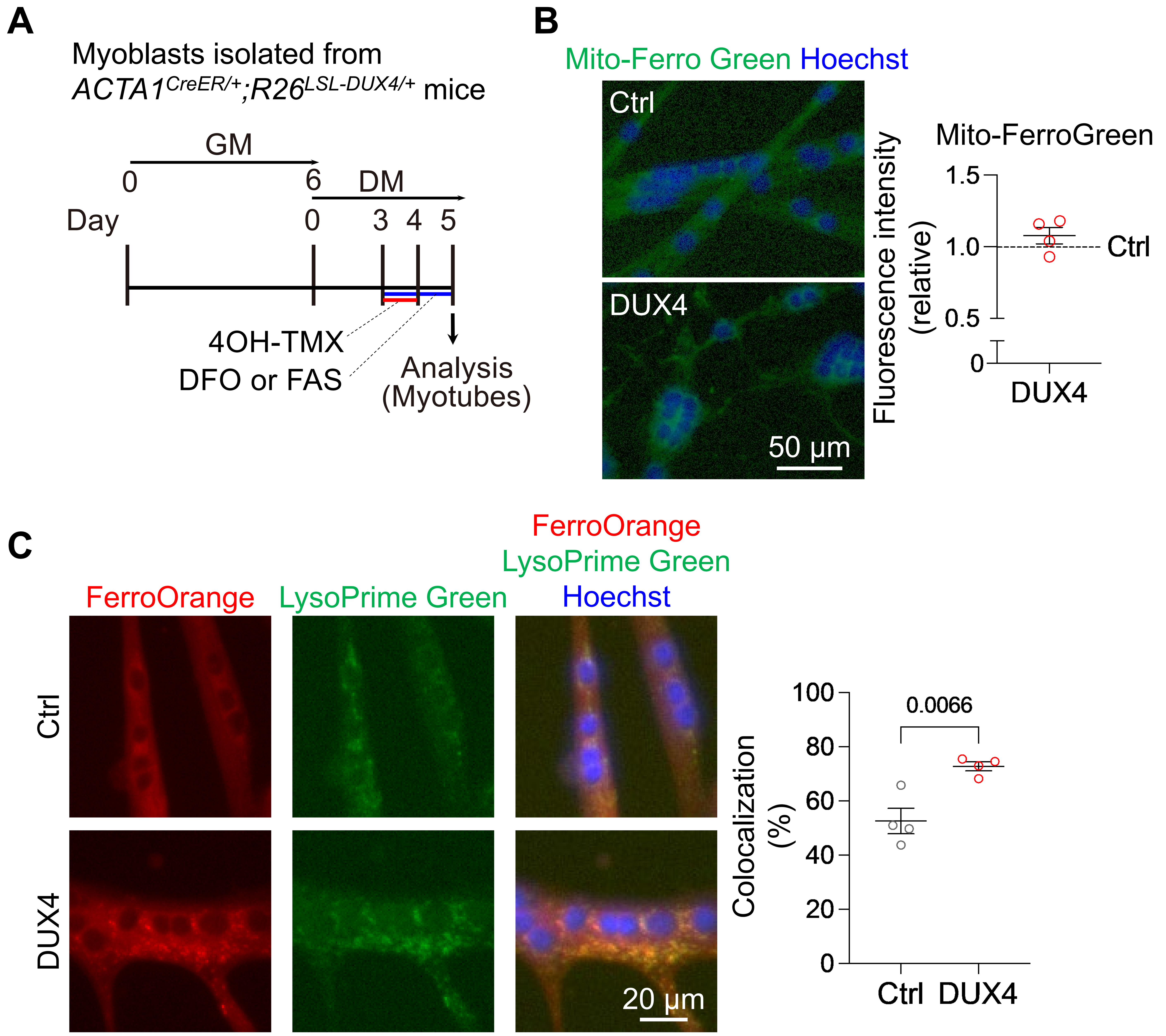

Supplementary Figure 9

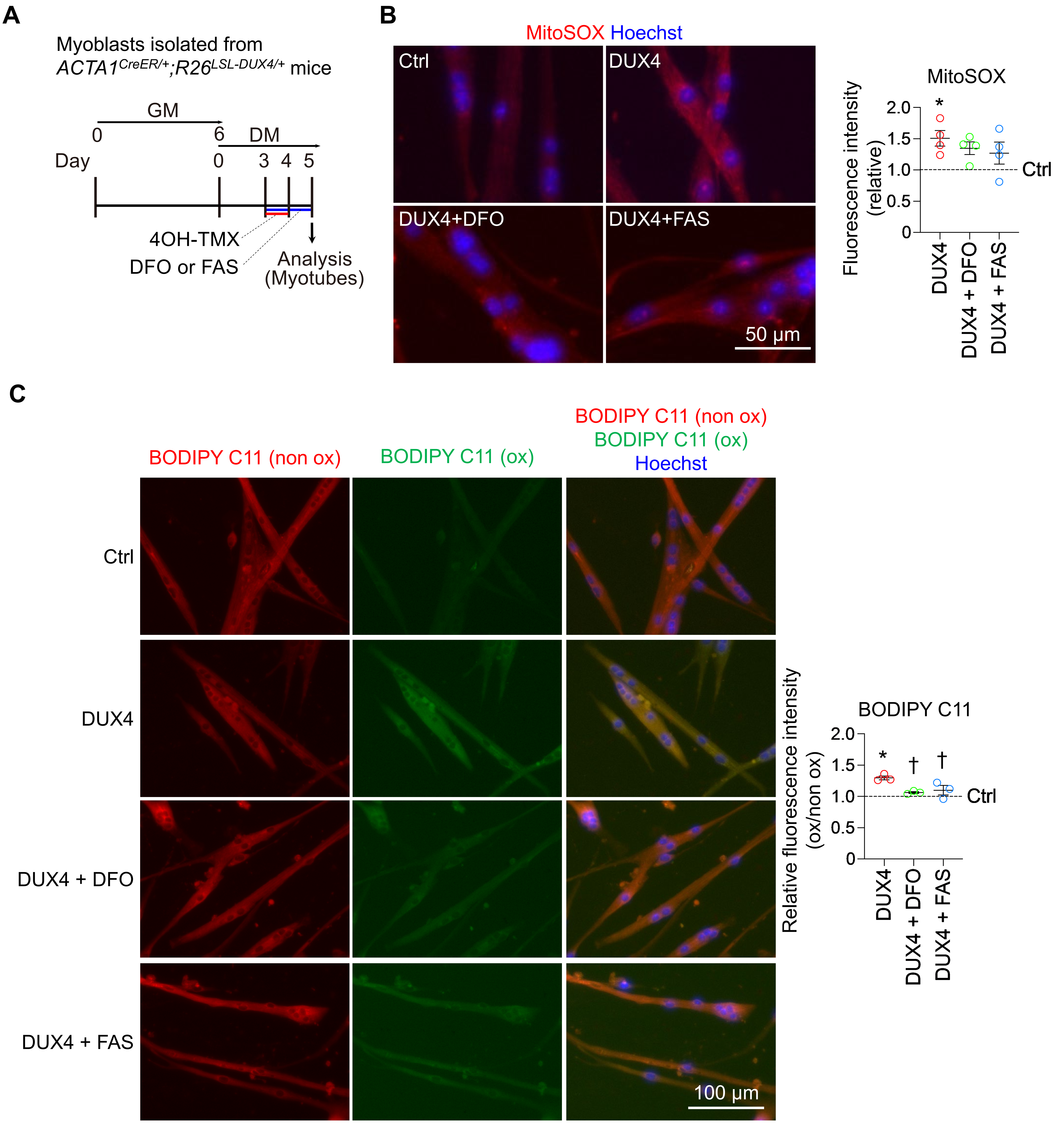

Supplement: Supplemental data [file jci-135-181881-s286.pdf]
