# Supplementary figures and images for: Iron supplementation alleviates pathologies in a mouse model of facioscapulohumeral muscular dystrophy
Source: J Clin Invest. 2025 Jul 1;135(17):e181881. doi: 10.1172/JCI181881 (PMC12404756; doi:10.1172/JCI181881)

Full unedited blot for Figure 8B

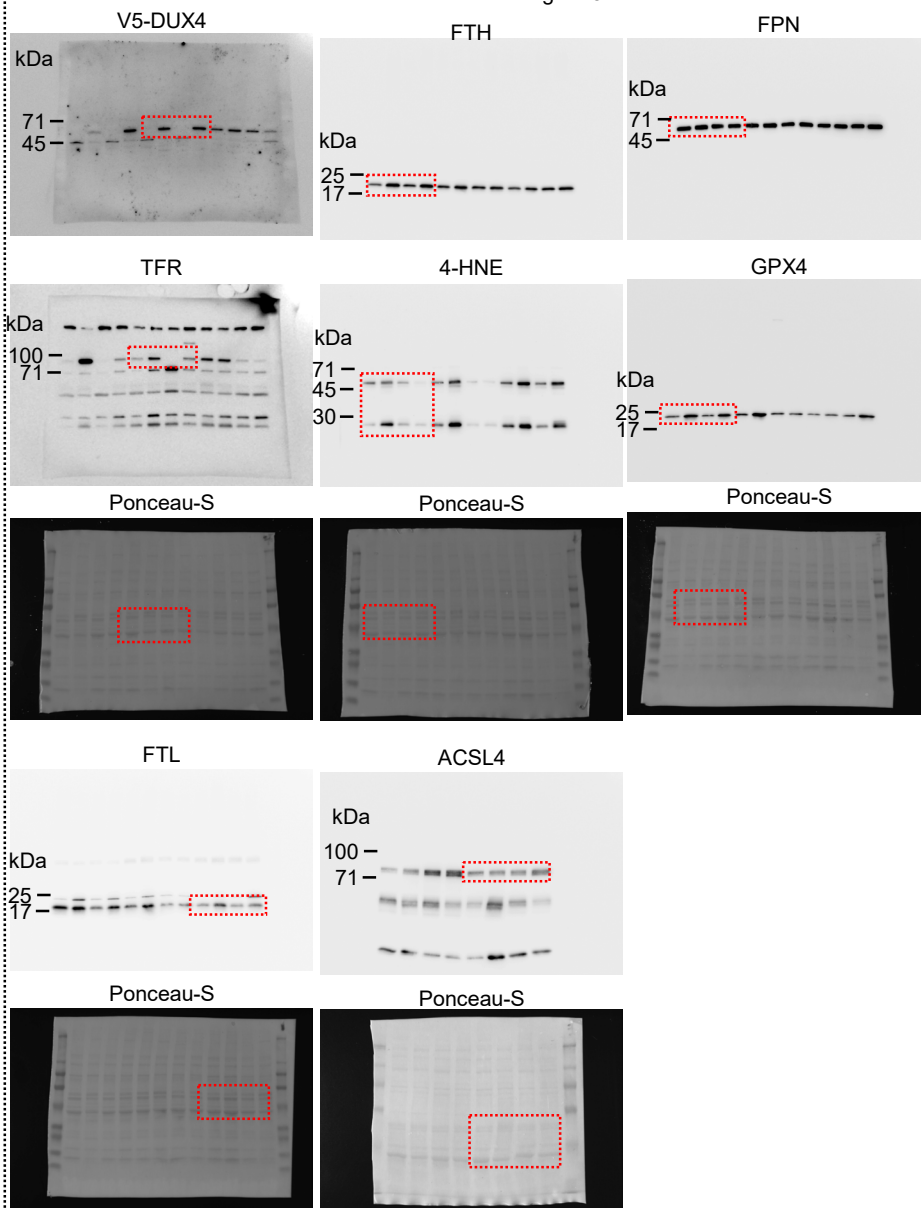

Full unedited blot for Supplementary Figure 7

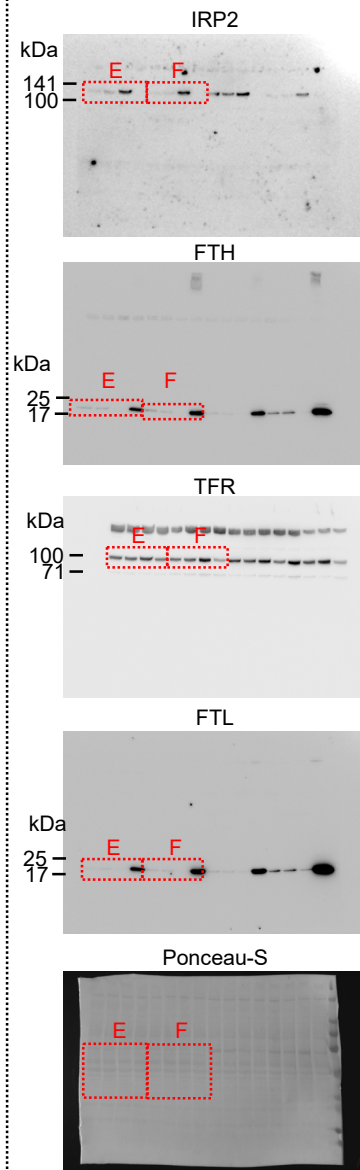

Supplement: Unedited blot and gel images [file jci-135-181881-s287.pdf]
